# Comparing two classes of biological distribution systems using network analysis

**DOI:** 10.1371/journal.pcbi.1006428

**Published:** 2018-09-07

**Authors:** Lia Papadopoulos, Pablo Blinder, Henrik Ronellenfitsch, Florian Klimm, Eleni Katifori, David Kleinfeld, Danielle S. Bassett

**Affiliations:** 1 Department of Physics & Astronomy, University of Pennsylvania, Philadelphia, Pennsylvania, United States of America; 2 Sagol School of Neuroscience, TelAviv University, Tel Aviv, Israel; 3 Department of Neurobiology, George S. Wise Faculty of Life Sciences, Tel Aviv University, Tel Aviv, Israel; 4 Department of Mathematics, Massachusetts Institute of Technology, Cambridge, Massachusetts, United States of America; 5 Mathematical Institute, University of Oxford, Oxford, United Kingdom; 6 Systems Approaches to Biomedical Science Doctoral Training Centre, University of Oxford, Oxford, United Kingdom; 7 Department of Physics, University of California San Diego, La Jolla, California, United States of America; 8 Section of Neurobiology, University of California San Diego, La Jolla, California, United States of America; 9 Department of Bioengineering, University of Pennsylvania, Philadelphia, Pennsylvania, United States of America; 10 Department of Electrical & Systems Engineering, University of Pennsylvania, Philadelphia, Pennsylvania, United States of America; 11 Department of Neurology, University of Pennsylvania, Philadelphia, Pennsylvania, United States of America; Harvard University, UNITED STATES

## Abstract

Distribution networks—from vasculature to urban transportation pathways—are spatially embedded networks that must route resources efficiently in the face of pressures induced by the costs of building and maintaining network infrastructure. Such requirements are thought to constrain the topological and spatial organization of these systems, but at the same time, different kinds of distribution networks may exhibit variable architectural features within those general constraints. In this study, we use methods from network science to compare and contrast two classes of biological transport networks: mycelial fungi and vasculature from the surface of rodent brains. These systems differ in terms of their growth and transport mechanisms, as well as the environments in which they typically exist. Though both types of networks have been studied independently, the goal of this study is to quantify similarities and differences in their network designs. We begin by characterizing the structural backbone of these systems with a collection of measures that assess various kinds of network organization across topological and spatial scales, ranging from measures of loop density, to those that quantify connected pathways between different network regions, and hierarchical organization. Most importantly, we next carry out a network analysis that directly considers the spatial embedding and properties especially relevant to the function of distribution systems. We find that although both the vasculature and mycelia are highly constrained planar networks, there are clear distinctions in how they balance tradeoffs in network measures of wiring length, efficiency, and robustness. While the vasculature appears well organized for low cost, but relatively high efficiency, the mycelia tend to form more expensive but in turn more robust networks. As a whole, this work demonstrates the utility of network-based methods to identify both common features and variations in the network structure of different classes of biological transport systems.

## Author summary

Distribution networks such as vasculature systems or urban transportation pathways are prevalent in our world. Understanding how different kinds of transport systems are organized to allow for efficient function in their environments and in the presence of constraints on material costs is currently an open area of investigation. In this study, we use methods from network science to compare and contrast the structure of two different classes of biological distribution networks: mycelial fungi and rodent brain vasculature. While each of these systems have been studied separately, less work has focused on understanding the diversity of their network organization. Here, we first examine several measures that characterize network connectivity on varying scales, finding that—although both systems have highly constrained network layouts—there are quantifiable differences in their architectures. Furthermore, using network analyses that specifically consider the embedding of these transport networks into real space, we observe that the two types of systems display distinct tradeoffs in network correlates of material cost, efficiency, and robustness. Together, our results provide evidence that while different distribution networks have general resemblances, they also exhibit variable design features that could reflect differences in their functions, environmental conditions, or development.

## Introduction

Transport networks—which are a subset of complex networks commonly studied using methods from network science [1]—represent structures throughout which entities are transferred between different regions of the system. Such networks are prevalent in both the engineered and natural world. One classic example is an urban transit system, where stations correspond to network nodes and where physical routes, such as roads or railways, correspond to network edges along which traffic can flow [2–4]. Examples from biology include vasculature networks, which allow for the distribution of blood to various parts of an organism, or collections of neurons and larger-scale brain areas connected by physical pathways, which allow for the transmission of electrical signals throughout the network. Importantly, all of these networks are *spatial* in the sense that the nodes and edges are embedded into real space [5]. The physical nature and spatial embedding of these systems often imposes costs associated with building and maintaining network infrastructure, and these costs can in turn constrain the network’s topology [6], for example, by making spatially long-distance connections improbable or by constraining the density of connections in the network. On the other hand, pressures that may compete against wiring minimization include those driving network efficiency and robustness. Tradeoffs between these desirable network features can vary across systems, and a quantitative understanding of such tradeoffs may directly inform the design of optimal spatial transport networks [7–14].

In this study, we focus on characterizing the network organization of biological distribution systems, which are indeed subject to the competing pressures of maintaining low material costs while achieving high efficiency and robustness. However, not all biological distribution systems are the same. Some can constitute an entire organism—such as mycelial fungi—in which the physical cords making up the organism can be represented as edges in a network, and in which branching, fusion, or end points among those cords can be represented as nodes in a network [15–19]. Past work has shown that these systems appear to strike an intermediate balance between cost and efficiency, enabling the organism to achieve competing goals. In addition, their network architecture can change and adapt over time in ways that can strengthen beneficial features, such as increased formation of cross-links and loops that aid in robustness to damage and allow for parallel flow pathways [15, 16, 18, 20–24]. Alternatively, distribution systems can form only a small part of a larger organism—as is the case with cortical vasculature—in which a pial network on the surface of the brain routes blood to penetrating arterioles, that in turn supply the underlying tissue [25, 26]. This vasculature system can also be modeled as a network, in which edges represent surface vessels, and nodes represent branching points among vessels or penetrating arterioles [27] that connect to and source an underlying three-dimensional system of microvessels. Previous investigations have found that the pial network of the middle cerebral artery in rodent brains forms a robust foundation of interconnected loops that can withstand damage and re-route flow in the presence of occlusions [27, 28].

In both the mycelial and vasculature systems studied here, planar distribution networks—whose nodes are distributed in two-dimensional space—must transport fluid and nutrients efficiently in the face of constraints on the total amount of material that they can support. But in spite of these commonalities, the two networks exist and have evolved in inherently different environments, which may directly impact the sorts of evolutionary pressures that the different networks experience. For example, the main role of the surface vasculature network is to transport blood to tissue that is part of a larger organism. On the other hand, for mycelial fungi, the network is the organism itself and is not necessarily constrained to serve or occupy a set region of space. Moreover, brain vasculature resides in a controlled environment within the confines of the skull, whereas mycelial networks live in and must adapt to often unprotected and varied environmental conditions [17, 29–31]. In addition, while directed flow and growth are known to be important in both vasculature and fungi, the mechanisms behind long-distance transport of nutrients and maturation are different in the two systems [17]. However, most past work has focused on characterizing these two classes of distribution networks separately from one another (see, for example, [25–27] and [15, 17, 20]), with little attention paid to how the varying habitats, function, and development of vasculature *vs.* mycelial systems may directly affect their network architectures. Thus, in contrast to prior studies, here we carry out a comparative analysis to make progress in understanding the similarities and differences in network organization across these two classes of natural transport systems.

To quantitatively compare and contrast the mycelial fungi and vasculature, we consider the binary network of nodes and edges that represents the structural backbone of connectivity underlying each system. We then utilize a set of methodologies rooted in network science to examine the organization of those networks. We first investigate the mycelial and rodent brain vasculature systems using a set of measures that probe different aspects of network topology, from those that evaluate local connectivity to those that assess hierarchical organization. We find that the two types of distribution networks exhibit some similarities, but also many differences in regard to certain network features across varying topological and spatial scales, despite the fact that both kinds of systems yield highly constrained, planar network layouts. In the second part of our study, we carry out a network-based analysis that purposefully takes into account the importance of the spatial embedding of the distribution systems, and we examine network correlates of properties that are more directly relevant to the function of these systems. In particular, by comparison to a set of spatially-informed null models, we examine relationships between network measures of wiring length, transport efficiency, and robustness, and compare and contrast the associated tradeoffs across the different network classes. This analysis uncovers some clear distinctions in how the two types of distribution networks balance competing goals, which we hypothesize may be markers for the systems’ functions or reflect the environment in which those functions must be performed. Taken together, our work demonstrates the utility of network science for characterizing and distinguishing different kinds of transport networks in biology, and underscores the fact that different systems can exhibit resemblances as well as important variations in their network structure. We hope that this study can inform future modeling and empirical work in this area, and that it provides a useful entry for more complex comparative investigations that consider both connectivity as well as edge weights (vessel/cord radii), in systems where this information is known.

## Materials and methods

### Data

#### Mycelial networks

Progress in image processing and analysis has allowed for automated extraction of digitized networks from images of mycelium [17]. In this study, we examine such networks from three different species. For a clean comparison against the rodent vasculature, we study a subset of networks that were ungrazed and grown without any additional resources aside from source innocula. In addition, since the vasculature was sampled at only one time point, for each type of mycelium, we use the most mature (i.e., the oldest) networks. Finally, we only consider networks with at least 500 nodes, so as to obtain good estimates of Rentian scaling (see Network measures). In all, we examine 4 different networks formed by *Phallus impudicus (P.I.)* grown from a single inoculum and sampled at 46 days, 3 networks from *Phanerochaete velutina (P.V. 1)* grown from 5 inocula and sampled between 30 days and 35 days, 5 networks from *Phanerochaete velutina (P.V. 2)* grown from 1 inocula and sampled between 39 days and 46 days, and 1 network from *Resinicium bicolor (R.B.)* grown from a single inoculum and sampled at 31 days. All of the data for the fungal networks was collected from several studies and made available online [19]. More information on details of these networks can be found in [19], the references therein, and in the documentation within the database.

#### Rodent vasculature networks

The rodent vasculature dataset consists of the pial networks in the region of the middle cerebral artery from four rats and five mice. To map out the networks, the vasculature was imaged and then traced by hand (further details can be found in the original study [27]). The rodent vasculature networks were kindly provided by Pablo Blinder and the group of David Kleinfeld at the University of California, San Diego.

### Network representations

In order to study the vasculature and mycelia using methods from network science, we first construct an undirected and unweighted adjacency matrix **A** for each network under consideration, with elements defined as

$$\begin{array}{c} A_{ij}=\{\begin{array}{cc} 1 & \text{if}\;\text{there}\;\text{is}\;\text{an}\;\text{edge}\;\text{between}\;\text{nodes}\;i\;\text{and}\;j,\\ 0 & \text{otherwise}. \end{array} \end{array}$$ (1)

This yields a binary connectivity matrix that captures the topological structure of the underlying system.

**Fig 1:**
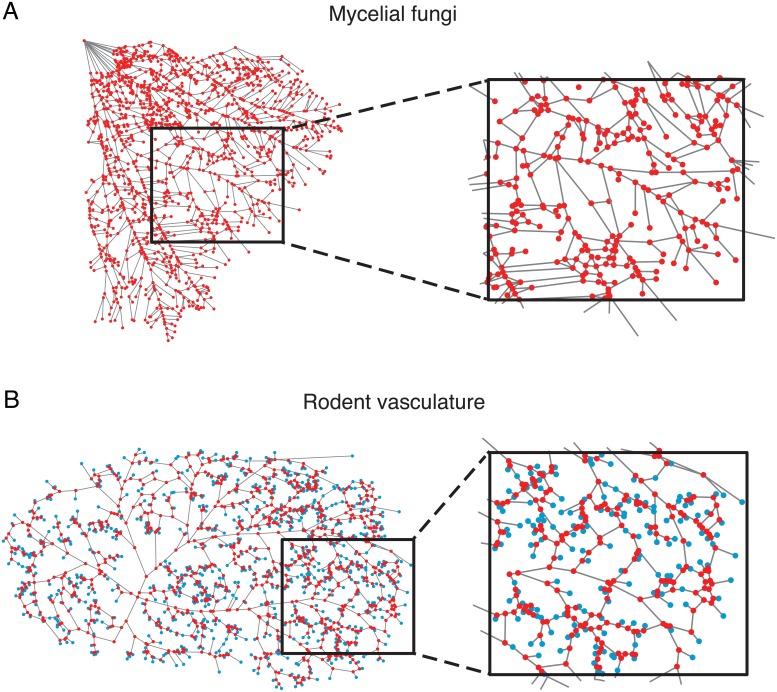
Distribution networks from mycelial fungi and rodent vasculature. *(A)* An example of a network from the fungus *Phallus impudicus* (*P.I.*). The cords making up the mycelial network are represented as edges (gray lines) and connect to form branching, fusion, or end points (red nodes). The top left node is the inoculum. *(B)* An example of a vasculature network from the surface of a rat brain in the region of the middle cerebral artery. Vessel segments are represented as edges (gray lines) and connect to form branching points on the surface backbone (red nodes) or connect to penetrating arterioles (blue nodes). In both *(A)* and *(B)*, the figures on the right are magnified sections of the full networks.

For the mycelial systems, the physical cords making up the organism are assigned to edges of the network represented in the adjacency matrix **A**, and the branching, fusion, or end points among those cords (as well as the inocula) are represented as nodes in the network. The networks describing the mycelia are 2-dimensional and planar, and all nodes have corresponding spatial coordinates in 2D. An example of an unweighted network from *P.I.* is shown in Fig 1A. Red points correspond to network nodes and gray lines correspond to the location of edges. See Fig. A in the S1 Text for additional examples of the unweighted network representations from the other species of mycelial fungi.

For the vasculature systems, edges of the network represented in the adjacency matrix **A** correspond to the location of vessel segments through which blood flows, and nodes are either junctions where surface vessels merge, or are penetrating arterioles that dive into and source the underlying neocortical microvasculature. In this study, we include both types of nodes in the network representation so that we can account for the vessels forming the surface backbone of loops as well as the many vessels that branch off the backbone and lead to penetrating arterioles, both of which are important functionally [27]. Furthermore, modeling the full network rather than just the subset of edges in loops allows for a fairer comparison with the mycelial systems, and a more complete quantification of network wiring lengths and other network measures involving pathways of edges. The vasculature networks are also 2-dimensional and planar, and all nodes have 2D spatial coordinates. We note that vessel radii information was not available for this data set, so we therefore focus on characterizing and comparing the unweighted network connectivity. An example of the entire vasculature network of the middle cerebral artery from a rat brain is shown in Fig 1B. Red nodes are branching points among the surface vessels and blue nodes are penetrating arterioles; gray lines represent the locations of edges. See Fig. B in the S1 Text for an example vasculature network from a mouse brain. In Table 1, we give the number of nodes (*N*) and number of edges (*M*) in each network.

**Table 1:** The number of nodes and edges for all studied networks. The first grouping corresponds to the mycelial networks and the second grouping corresponds to the vasculature networks.

Network type	Number of nodes (*N*)	Number of edges (*M*)
P.I. 1	1357	1858
P.I. 2	543	725
P.I. 3	1029	1317
P.I. 4	1519	2057
P.V.1 1	1212	1564
P.V.1 2	1384	1851
P.V.1 3	1209	1527
P.V.2 1	986	1351
P.V.2 2	551	627
P.V.2 3	553	670
P.V.2 4	948	1088
P.V.2 5	1204	1468
R.B.	602	850
Rat 1	1712	1823
Rat 2	2043	2168
Rat 3	2158	2296
Rat 4	2650	2775
Mouse 1	826	855
Mouse 2	1449	1481
Mouse 3	672	700
Mouse 4	967	994
Mouse 5	947	973

It is important to note that because transport systems are physical in nature, there is often additional information that can be incorporated into analyses due to the embedding of their networks into real space. This is useful to consider, because in such spatial networks, the connectivity and the geometric layout of network elements are likely to be interwined and constrained by one another. The networks studied here are embedded in two dimensions, so a given node *i* has a spatial coordinate, {*x*_*i*_, *y*_*i*_}, which allows the Euclidean distance between nodes *i* and *j*, *D*_*ij*_, to be computed. In reality, the cords or vessels also have some radius *r*_*ij*_, and one could thus construct a weighted network representation of the system, in which edges of the corresponding adjacency matrix are weighted by a function of both length and radius [15, 17, 19–21, 25, 26]. However, since cross-sectional area information was not available in the rodent vasculature experiments, we consider only the edge length in this study. Although the spatial coordinates of nodes and edge lengths do not encompass the full geometric structure of the underlying system, they still capture important information about how the system is laid out in space. Furthermore, in conjunction with the network connectivity, incorporating spatial information about inter-node distances allows one to estimate quantities such as wiring length and more relevant assessments of network efficiency (see Network measures for details). These kinds of metrics complement and augment network-based analyses that consider only network topology with no regard to spatial embedding. However, we do acknowledge that radial information is crucial for understanding distribution systems, and this will be important to include in future work.

### Network measures

Network science provides a powerful mathematical foundation for the principled study of diverse complex networks. Within this framework, there are many different methods and metrics that one can use to quantify network properties. Below (and in more detail in the S1 Text), we describe the measures utilized in this study.

#### Standard topological graph metrics

In our analysis, we begin by first characterizing the different distribution networks using a set of widely-utilized graph metrics that quantify the connectivity—or topology—of a network, without utilizing information about the physical locations of nodes and edges. In particular, we will examine the *mean degree* 〈*k*〉, the *average clustering coefficient* *C*, the *alpha index* *α*, the *topological efficiency* *E*^*t*^, and the *topological edge betweeness centrality* $B_{e}^{t}$. In short, the mean degree measures the average number of edges incident to nodes in a network, the clustering coefficient quantifies the occurrence of fully connected triplets of nodes in a network, the alpha index measures the density of elementary cycles of all lengths in a planar network, the topological efficiency is defined as the average of the inverse of the shortest topological path lengths between all pairs of network nodes, and the topological edge betweenness centrality measures the number of shortest topological paths that go through a given edge. As these are widely used measures in the network science literature, we leave more detailed descriptions and definitions to the S1 Text.

#### Rentian scaling

We also investigate *Rentian scaling* [32–37] in the distribution systems, which is an empirical scaling relationship between the number of nodes *n* in a partition of a network and the number of edges *m* crossing the boundary of that partition, such that *m* ∝ *n*^*r*^ where 0 ≤ *r* ≤ 1 is the Rent exponent. Since its initial discovery in very large scale integrated (VLSI) circuits [38], this phenomena has been observed in biological neural systems [39, 40] and in urban transportation networks [3]. As described further below, Rentian scaling can be examined in both the topological and physical space of a network, and the presence of Rentian scaling indicates a type of hierarchical organization and can be used to assess network complexity.

*Topological Rentian scaling* is indicated by a relationship of the form, *m* ∝ *n*^*t*^, where *n* is the number of nodes inside a topological partition of the network, *m* is the number of edges crossing the partition boundary, and 0 ≤ *t* ≤ 1 is the topological exponent. More specifically, one asks whether this relationship holds across partitions of varying sizes. Here, we assess networks for topological Rentian scaling using the hyper-graph partitioning package *hMETIS* [41, 42], in which a recursive min-cut bi-partitioning algorithm recursively splits the network into halves, quarters, etc. in a way that attempts to minimize the number of edges passing from one partition to another (see Fig. D(i) in the S1 Text). After each round of partitioning, we track the number of nodes *n* in each partition, and the number of edges *m* crossing the partition boundaries; if the relationship between these quantities obeys the form *m* ∝ *n*^*t*^, then the network is said to show topological Rentian scaling, and we can estimate the scaling exponent *t* from the slope of a best-fit line through log *m* *vs.* log *n* (Fig. D(ii) in the S1 Text). See the S1 Text for further details on how this analysis is carried out. Rather than considering *t* in isolation, it is also useful to recall the relationship between topological scaling and network complexity, as developed in VLSI theory. In particular, the topological dimension of a network *d*_*T*_ is related to *t* via $t\geq 1-\frac{1}{d_{T}}$; thus, higher topological exponents indicate higher dimensional network topologies [33, 36].

The presence of *physical Rentian scaling* can be examined by testing for a relationship of the form *m* ∝ *n*^*p*^, where *m* is the number of network edges crossing the boundary of a contiguous physical partition of the network, *n* is the number of nodes inside the partition, and *p* is the physical Rent exponent. More specifically, one examines whether this relationship holds over many partitions across different length scales in real space. To empirically test for this relationship in a network, we consider 5000 square partitions of the network, where the side length and center position of each partition is chosen at random (see Fig. D(iii) in the S1 Text). In order to avoid boundary effects due to the finite size of the network, we only sample boxes contained within the network’s convex hull. We then compute the number of nodes *n* inside each partition and the number of edges *m* crossing the partition boundary (Fig. D(iv) in the S1 Text), and test whether these quantities are related by a power law of the form *m* ∝ *n*^*p*^, where *p* is the physical Rent exponent (see Fig. D(v) in the S1 Text). If such a relationship holds, then the exponent *p* can be estimated from the slope of a best-fit line through log *m* *vs.* log *n* (see S1 Text for more details on how this analysis is carried out). The existence of physical Rentian scaling signifies hierarchical structure in the spatial layout of the network, and furthermore, because the scaling is determined from a random sampling of the network, its existence signifies a degree of spatial homogeneity and space-filling capacity. Importantly, physical Rentian scaling extends ideas of self-similarity (such as the fractal dimension [43]), which are agnostic to the spatial positioning of network elements. Moreover, the theoretical minimum value for *p* is related to the Euclidean dimension *d*_*E*_ into which a network is embedded and the topological Rent exponent *t* via $p^{\text{min}}=\text{max}\left(1-\frac{1}{d_{\text{E}}},t\right)$ [34, 39]. The theory of VLSI circuit design predicts that low values of *p* (i.e., values close to the theoretical minimum) are associated with networks that have been cost-efficiently embedded into physical space, in terms of wiring length [32, 34, 39].

#### Biophysically-motivated network measures

In addition to standard measures of network topology, we can also compute measures of network organization that are more directly related to the functional requirements of and constraints on biological distribution networks. We describe these biophysically-motivated network metrics in the remainder of this section.

#### Wiring length

For spatially embedded networks such as the transport systems considered in this study, one can approximate the total wiring length of the networks, which in turn provides an estimate of material cost. Given a distance matrix *D*_*ij*_ that encodes the Euclidean distance between all pairs of nodes, and an undirected, unweighted adjacency matrix *A*_*ij*_ that represents network connectivity, we define the total wiring length of the network as

$$\begin{array}{c} W=\sum_{i>j}A_{ij}D_{ij}. \end{array}$$ (2)

Note that this definition of wiring length assumes that vessels or cords are straight, and neglects additional wiring length that may result from tortuosity.

#### Physical efficiency

For spatial networks, we can also examine paths along network edges using physical distances, rather than topological distances. In particular, the shortest physical path between a pair of nodes *i* and *j* is taken to be the path that minimizes the sum of the lengths of the edges traversed in a path connecting nodes *i* and *j*. This is in contrast to the shortest topological path between the same nodes, which neglects geometric information. Using physical path lengths rather than topological path lengths, we can compute the average *physical efficiency* $E_{\text{avg}}^{p}$. This quantity is defined as

$$\begin{array}{c} E_{\text{avg}}^{p}=\frac{1}{N(N-1)}\sum_{i,j}\frac{1}{l_{ij}^{p}}, \end{array}$$ (3)

where $l_{ij}^{p}$ is the length of the shortest physical path along network edges between nodes *i* and *j*. Dividing $E_{\text{avg}}^{p}$ by the corresponding value for a fully connected network with the same number of nodes yields the global physical efficiency *E*^p^, where 0 ≤ *E*^p^ ≤ 1. Shortest path and efficiency measures that explicitly incorporate the geometric layout of a network have previously been used to study spatial networks such as ant galleries [44], street patterns in cities [45, 46], brain networks [47], slime mould [48], and fungal networks [17, 20–23], but to the best of our knowledge, have not yet been used to quantify animal vasculature networks.

#### Physical edge betweenness centrality

From the shortest physical paths between all node pairs, we can also calculate a *physical edge betweenness centrality* $B_{e}^{p}$. Letting $\frac{\psi_{qr}^{p}\left(i,j\right)}{\psi_{qr}^{p}}$ be the fraction of shortest paths (measured in physical units of distance) between *q* and *r* that pass through edge (*i*, *j*), we compute

$$\begin{array}{c} B_{e}^{p}\left(i,j\right)=\sum_{q,r}\frac{\psi_{qr}^{p}\left(i,j\right)}{\psi_{qr}^{p}}. \end{array}$$ (4)

For an edge (*i*, *j*), this quantity is thus the fraction of shortest physical paths between all node pairs that traverse the edge (*i*, *j*).

#### Network robustness

Finally, we note that a desirable feature for both natural and artificial networks is robustness. Here, we consider structural robustenss in the sense of how well-connected a network remains when subjected to damage. We probe this by removing a fraction of the total number of edges at random from a network, and then track how the size of the largest connected component evolves as the fraction of removed edges increases. In particular, we define the robustness *R* of a network to be the percentage of edges removed in order for the size of the largest connected component to drop to half of its original value [44, 45]. Since edges are removed at random, there can be some variation in the results when the same procedure is run again on the same network. We thus compute *R* a total of 20 times for each network in order to generate a representative ensemble average. We again note that in our analysis, we consider the robustness of the entire vasculature network, rather than just the backbone, as considered in [27]. This allows for a cleaner comparison between the vasculature and fungi, and takes into consideration the many vessels that branch directly off of the backbone to penetrating arterioles.

### Null models

In order to place various measures of network organization into context, or to compare and contrast empirical networks of varying size, it is useful to normalize network properties computed on a given network G by their corresponding values in null model networks that have the same number of nodes as G. Null models are often canonical networks that preserve certain features of the original network (e.g., density) or are networks that have idealized or extreme topological and/or spatial organization (e.g., lattice-like, random, or fully connected) [49].

#### Randomly-rewired benchmarks

One null model we consider is a type of random graph—sometimes called the *configuration model*—constructed by randomly rewiring a given empirical network while preserving its degree distribution. To generate these randomized benchmarks, we use the Brain Connectivity Toolbox [50], which contains a function based on the algorithm introduced in [51], and that also maintains the connectedness of the rewired network. Each edge is rewired approximately 15 times. This null model is employed in the first part of our analysis, in order to better compare certain topological metrics across the set of empirical distribution networks. In particular, for each empirical network, we generate an ensemble of 10 randomly rewired networks. We then compute the mean value 〈*X*_rewire_〉 of a given metric across the ensemble of randomly rewired networks, and normalize the empirical value *X* by the reference value 〈*X*_rewire_〉. We can then compare the normalized values $\frac{X}{\langle X_{\text{rewire}}\rangle }$. This allows us to ask if different kinds of distribution networks exhibit differences in specific network properties, while controlling for variability in network size and degree distribution. Note that while this null model preserves certain topological features of an empirical network, it is agnostic to spatial features.

#### Spatial null models for wiring, efficiency, and robustness

In spatial networks, the nodes and edges exist in real space, and this physical embedding often has significant consequences for the network topology [5]. An important constraint for biological systems in particular is the material and energetic costs associated with building and maintaining the physical structure of the network. But a competing constraint stems from the fact that distribution networks must be able to move resources efficiently and be robust to damage. In order to gain an understanding of how the spatial embedding of these networks might affect their architecture, and how distinct types of biological transport systems may differentially balance certain pressures, in the second part of our analysis we compare the empirical networks to two idealized planar null model networks: the minimum spanning tree (*MST*) and the greedy triangulation (*GT*). The same or similar null models have previously been used in the analysis of fungal systems, [17, 20–23], slime mould [48], networks of ant galleries [44], and urban street networks [45, 46].

**Fig 2:**
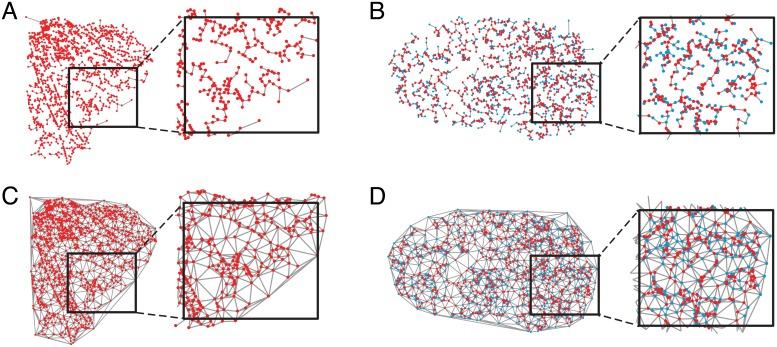
Construction of spatial null model networks. *(A,B)* The minimum spanning tree *MST* and *(C,D)* the greedy triangulation *GT* for the mycelial network and vasculature network shown in Fig 1.

The *MST* is a graph that connects all of the nodes in a network such that the sum of the total edge weights is minimal. By construction, the *MST* also contains the minimum number of edges *M* needed to connect a network (*M* = *N* − 1, where *N* is the number of nodes). In the biological distribution networks studied here, the relevant edge weights are their physical lengths. Therefore, for the *MST* null model, we preserve the true spatial locations of all nodes in the empirical network, and compute the *MST* on the matrix of Euclidean distances, *D*_*ij*_, between all node pairs. The resulting network is planar and minimizes the total wiring length *W* of the network (Eq 2), given the spatial distribution of nodes. The *GT* represents the opposite extreme, and is a maximally connected—in terms of the number of edges—planar network. In particular, following [44–46], we compute *GT*s on the true node locations of all real networks by iteratively connecting pairs of nodes in ascending order of their distance while ensuring that no edges cross. As with the *MST*, this null model is also constructed under a geometric constraint, but since it contains many more edges, it represents an effective upper bound on the wiring length of a planar network with given node locations. In Fig 2, we show the *MST* and *GT* for the mycelial and vasculature networks depicted in Fig 1.

#### Relative measures of wiring length, spatial efficiency, and robustness

In the second part of our analysis, we examine a set of network measures that explicitly take into account spatial constraints and that are more directly pertinent to the function of transport networks. These are the wiring length, physical efficiency, and robustness (see Biophysically-motivated network measures). It is important to note that these kinds of metrics have previously been used to quantify and compare the structure of mycelial networks [17, 20–23]. Here, we will build on and extend prior analyses by examining tradeoffs and relationships between these quantities, and most importantly, we focus on comparing the wiring length, network efficiency, and structural robustness across the vasculature and mycelial networks.

While useful measures to consider in the context of distribution systems, when considered in isolation, it is difficult to understand how these metrics are related to one another and how they compare across similar networks of different size or of completely different type. In order to better understand how the network architecture of the vasculature and mycelial systems might be differentially organized for each of these measures, we thus use a set of normalized quantities that characterize how the wiring length, efficiency, and structural robustness of a given empirical network compare to approximate limiting values for a network of the same size and with the same node locations. In particular, following [44–46], we define a *relative* cost, physical efficiency, and structural robustness using the *MST* and *GT* null models.

The relative cost is

$$\begin{array}{c} W_{\text{rel}}=\frac{W-W_{\text{MST}}}{W_{\text{GT}}-W_{\text{MST}}}, \end{array}$$ (5)

where *W*, *W*_MST_, and *W*_GT_ denote the total wiring length of the real, *MST*, and *GT* networks, respectively. In a similar manner, the relative global physical efficiency, $E_{\text{rel}}^{p}$, is given by

$$\begin{array}{c} E_{\text{rel}}^{p}=\frac{E^{p}-E_{\text{MST}}^{p}}{E_{\text{GT}}^{p}-E_{\text{MST}}^{p}}, \end{array}$$ (6)

and the relative robustness *R*_rel_ is

$$\begin{array}{c} R_{\text{rel}}=\frac{R-R_{\text{MST}}}{R_{\text{GT}}-R_{\text{MST}}}. \end{array}$$ (7)

By definition, the *MST* is minimally wired, but since it contains no redundancy or shortcuts, we also expect it to have low efficiency. It is also clear that the removal of any edge will break the *MST* into disconnected components. In contrast, the *GT* is a highly expensive network to build, but this increase in network density for the same set of nodes should improve the robustness as well as the efficiency of the network, since it allows for shortcuts between pairs of otherwise more distant nodes. Thus, the *MST* is an effective lower bound for wiring length, efficiency and robustness, and the *GT* is a good approximation for a planar network that achieves upper bounds with respect to the same measures. The relative measures are normalized between 0 and 1 for the *MST* and *GT*, respectively. This normalization allows for a direct quantification of where the real distribution networks lie in comparison to the limiting values achievable for a fixed spatial distribution of nodes. Furthermore, the relative quantities can be used to understand and properly contrast the tradeoffs between wiring, efficiency, and resistance to random damage between the vasculature and fungi.

### Statistical comparisons

In order to make statistical comparisons between the two types of distribution networks—mycelial fungi *vs.* vasculature—we first group all mycelial networks together and all vasculature networks together, and use two-sample *t*-tests to compare network measures between the two groups. Statistically significant differences between the two groups with respect to the mean value of a particular measure (which we denote using an overbar symbol and subscripts “F” or “V” for fungi and vasculature, respectively) are indicated by a *p*-value < 0.05. Because there are only a small number of networks in the individual subgroups of fungi (i.e., P.I., P.V. 1, P.V. 2, R.B.) and vasculature (i.e., rat, mouse), rather than making comparisons at the level of each subgroup—which would not permit a robust statistical analysis—we use the method of first grouping the data and then examining overall differences or similarities between the broad classifications of network type.

## Results

We now examine the vasculature and mycelial networks using the metrics and null models outlined in the previous section. The overarching goals of the following analysis are to characterize the network structure of the two types of distribution systems, and to compare and contrast their network architectures. Our study is decomposed into two main sections: Characterization of network architecture with graph-theoretical measures and Comparative analysis of network organization using biologically-motivated measures. In the first section, we begin by considering a set of standard graph-theoretical metrics that describe topological features of a network (i.e., those which are connectivity-based and that can be computed regardless of a spatial embedding). The different measures within this set can be classified based on the topological scale they assess, ranging from, for example, degree (which quantifies structure in the local neighborhood of nodes) to topological efficiency (which probes network organization on a more global scale). We will see that the vasculature and mycelia are distinguishable in terms of each topological graph metric considered. Next, we investigate the relationship between topological and physical edge-betweenness centrality, finding that the extent of the overlap between the spatial and non-spatial version of centrality differs across the two classes of distribution systems. Finally, inspired by notions of hierarchical and cost-efficient organization in transport networks more broadly, we close the first part of our analysis by examining topological and physical Rentian scaling in the vasculature and mycelia. The aforementioned analyses then lead into and motivate the second main portion of the results, in which we turn from a description of the two distribution networks with classic graph measures to a more biophysically-informed characterization of the vasculature and mycelia using the spatial null models and a set of biologically-inspired network measures that may be more directly relevant to the function of these systems. In particular, this latter analysis uncovers distinctions in how the two kinds of systems balance network-based estimates of material cost, efficiency, and robustness. For easy reference, the main results of our analyses are summarized in Table 2.

**Table 2:** Comparison of various network properties between the mycelial and vasculature networks. The first column indicates the network property. The second and third columns give the mean ± the standard error of the mean for each network property, averaged over the population of fungal networks and the population of vasculature networks, respectively. The last column indicates the level of significance from a two-sample *t*-test used to assess statistical differences in the mean values of each network property between the two types of distribution networks; **p-value <0.001, ***p-value <0.0001.

Network property	Mycelial fungi	Vasculature	Significance level
Mean degree 〈*k*〉	2.57 ± 0.05	2.09 ± 0.01	***
Normalized clustering $\frac{C}{\langle C_{\text{rewire}}\rangle }$	54.67 ± 7.88	6.44 ± 1.61	***
Alpha index *α*	0.144 ± 0.012	0.022 ± 0.003	***
Normalized topological efficiency $\frac{E^{t}}{\langle E_{\text{rewire}}^{t}\rangle }$	0.60 ± 0.02	0.84 ± 0.03	***
Spearman correlation between topological and physical edge betweenness centrality *ρ*	0.65 ± 0.03	0.94 ± 0.01	***
Normalized physical Rent exponent $\frac{p}{\langle p_{\text{rewire}}\rangle }$	0.609 ± 0.007	0.533 ± 0.002	***
Normalized topological Rent exponent $\frac{t}{\langle t_{\text{rewire}}\rangle }$	0.44 ± 0.01	0.59 ± 0.04	**
Ratio of theoretical minimum physical Rent exponent to true physical Rent exponent $\frac{p^{\text{min}}}{p}$	0.826 ± 0.007	0.949 ± 0.004	***
Relative wiring *W*_rel_	0.302 ± 0.013	0.080 ± 0.004	***
Relative physical efficiency *E*_rel_	0.43 ± 0.03	0.36 ± 0.03	—
Relative robustness *R*_rel_	0.38 ± 0.02	0.15 ± 0.01	***

### Characterization of network architecture with graph-theoretical measures

#### Standard network metrics

To begin, we characterize the vasculature and mycelia using a series of standard topological network metrics.

**Fig 3:**
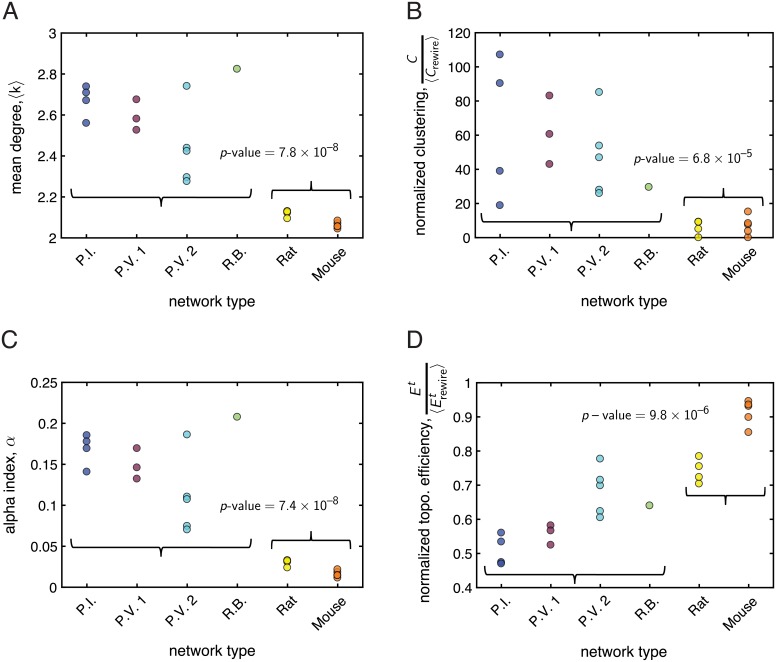
Comparison of topological network metrics between the mycelial and vasculature systems. In each panel, the *x*-axis labels the kind of network, with the curly braces grouping the mycelia (first 4 networks) and vasculature (last 2 networks). The *y*-axis measures the value of a given property for each network. A *p*-value is displayed if we found a statistically significant difference (as determined by a two-sample *t*-test) in the mean value of the given property between the population of mycelial networks and the population of vasculature networks. *(A)* The mean degree 〈*k*〉 was significantly larger in the fungi compared to the vasculature. *(B)* The normalized clustering coefficient $C/\langle C_{\text{rewire}}\rangle$ was significantly larger in the fungi compared to the vasculature. *(C)* The alpha index *α* was significantly larger in the fungi compared to the vasculature. *(D)* The normalized topological efficiency $E^{t}/\langle E_{\text{rewire}}^{t}\rangle$ was significantly larger in the vasculature compared to the fungi. See the main text for more details on each measure.

Focusing first on measures that assess local connectivity in the immediate neighborhood of nodes in a network, we found that the *mean degree* 〈*k*〉 was highly constrained, falling between 2 and 3 for all networks. This is expected for biological flow networks, in which junctions typically develop from the branching, fusion, or anastomosis of vessels or cords [17, 52]. However, even within this narrow range, we observed statistically significant differences in 〈*k*〉 for the vasculature *vs.* fungi (*p*-value = 7.8 × 10^−8^, Fig 3A), with the mycelial networks having larger mean degrees (${\bar{\langle k\rangle }}_{F}=2.57$) than the vasculature systems (${\bar{\langle k\rangle }}_{V}=2.09$). We also examined the *clustering coefficient*, which measures transitivity in a network (i.e., the density of the shortest possible cycles in a network—those of length three). Considering, in particular, the normalized clustering coefficient $C/\langle C_{\text{rewire}}\rangle$ (Fig 3B), we found that most of the empirical networks tended to have larger clustering relative to their randomly-rewired null models, although two of the vasculature networks had *C* = 0. We note that, especially for the vasculature, many instantiations of the randomly rewired networks also yielded clustering coefficients of zero, pointing out that triangles are generally rare in randomized networks of the same size and degree distribution. Nevertheless, in total across the two kinds of distribution systems, we found that the mycelial networks had higher normalized clustering coefficients ($\bar{C_{F}/\langle C_{\text{rewire}}\rangle }=54.67$) than the vasculature networks ($\bar{C_{V}/\langle C_{\text{rewire}}\rangle }=6.44$), and this difference was statistically significant (*p*-value = 6.8 × 10^−5^). This result indicates that compared to an ensemble of size and degree-distribution preserving null models, the mycelial networks tend to have a higher density of 3-edge cycles than the vasculature networks.

While the clustering coefficient assesses a network for the existence of cycles of length three, we can also consider loops (or cycles) on larger topological length scales. This is particularly relevant for distribution systems, as past work on empirical vasculature networks [27] and models of flow networks [53] have found that loops can serve to protect the system from occlusions or damage to veins. To quantify the presence of cycles of all lengths in the mycelial and vasculature networks, we computed the *alpha index* *α* [5, 45], which is equal to 0 for a network that is a tree (no cycles) and is equal to 1 for a maximal planar network. The alpha index is plotted for all networks in Fig 3C. One observes that the vasculature networks tend to have lower alpha indices ($\bar{\alpha_{\text{V}}}=0.02$) compared to the fungal networks ($\bar{\alpha_{\text{F}}}=0.14$), and we found that this difference was statistically significant (*p*-value = 7.4 × 10^−8^). Thus, compared to maximal planar networks of the same size, the mycelial networks have relatively more loops than the vasculature networks. As expected [5], the alpha index gives similar information to the mean degree.

We next examined the *topological efficiency* *E*^*t*^, which—in contrast to the previous metrics—evaluates larger-scale organization in a network by considering the shortest (topological) pathways between all pairs of nodes. To compare the topological efficiency across the vasculature and fungi, for each network we computed the normalized quantity $E^{t}/\langle E_{\text{rewire}}^{t}\rangle$ that measures how the global topological efficiency of each distribution network compares to a collection of rewired networks of the same size and degree distribution (Fig 3D). We found a statistically significant difference in the population averages of the normalized topological efficiencies of the vasculature *vs.* mycelial networks ($\bar{E_{\text{V}}^{t}/\langle E_{\text{rewire}}^{t}\rangle }=0.84$, $\bar{E_{\text{F}}^{t}/\langle E_{\text{rewire}}^{t}\rangle }=0.60$; *p*-value = 9.8 × 10^−6^). This result suggests that the two kinds of distribution networks can also be distinguished in terms of this measure of their global organizational structure.

**Fig 4:**
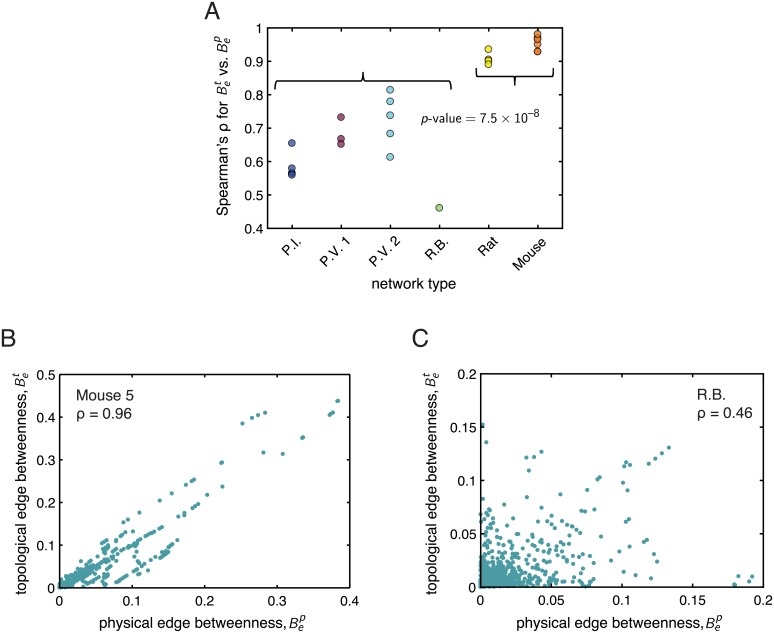
Topological *vs.* physical edge-betweenness centrality. *(A)* For each kind of network (displayed on the *x*-axis), the data shows the Spearman rank correlation coefficient *ρ* for the relationship between the topological edge betweenness centrality $B_{e}^{t}$ and the physical edge betweenness centrality $B_{e}^{p}$. The curly braces group the fungi (first 4 networks) and vasculature (last 2 networks). All networks exhibited significant positive rank correlations, but the displayed *p*-value from a two-sample *t*-test indicates that the mean rank correlation coefficient of the mycelial networks was significantly smaller than the mean rank correlation coefficient of the vasculature networks. *(B)* Topological edge betweenness centrality $B_{e}^{t}$ *vs.* physical edge betweenness centrality $B_{e}^{p}$ for the network with the highest Spearman correlation between these quantities (Mouse 5). *(C)* Topological edge betweenness centrality $B_{e}^{t}$ *vs.* physical edge betweenness centrality $B_{e}^{p}$ for the network with the lowest Spearman correlation between these quantities (R.B.). See the text for more details on these measures.

As previously noted, for spatially embedded networks, we can also measure path lengths in terms of Euclidean distances. An interesting question one can then ask is: how much overlap is there between counterpart physical and topological measures of network organization? Do they contain the same (or similar) information, or measure something distinct about the system? Here, we examine the relationship between the topological edge betweenness centrality $B_{e}^{t}$ and the physical edge betweenness centrality $B_{e}^{p}$, which measure the fraction of shortest topological and physical paths, respectively, that pass through a given edge in a network. For each distribution network, we computed the Spearman rank correlation coefficient *ρ* between $B_{e}^{t}$ and $B_{e}^{p}$ to assess the strength and direction of a monotonic relationship between the two quantities (see Fig 4A). Interestingly, while there was a significant, positive rank correlation between $B_{e}^{t}$ and $B_{e}^{p}$ for all networks, we also observed a clear and statistically significant difference (*p*-value = 7.5 × 10^−8^) in the average rank correlation coefficient of the population of mycelial networks compared to that of the vasculature networks. In particular, we found that the mycelial networks had smaller *ρ* values than the vasculature networks ($\bar{\rho_{F}}=0.65$, $\bar{\rho_{V}}=0.94$). Thus, in the vasculature networks, the topological and physical edge betweenness centralities contain similar information in that edges that participate in many of the shortest topological paths also participate in many of the shortest physical paths through the network. But this overlap is not nearly as strong in the mycelial networks, suggesting that topological and physical correlates of network architecture need not necessarily be redundant. Fig 4B and 4C show $B_{e}^{t}$ *vs.* $B_{e}^{p}$ for the networks with the highest (Mouse 5) and lowest (R.B.) Spearman correlations, respectively. These findings motivate the second major part of our analysis (Comparative analysis of network organization using biologically-motivated measures), in which we characterize and compare and contrast the vasculature and mycelial networks using spatial and more biologically-motivated measures of network structure that take into account the physical nature of these systems.

#### Rentian scaling

Important questions regarding biological transport systems are whether distribution networks of different types or from different organisms exhibit universal characteristics that allow for high functionality, and in particular if and how they achieve embeddings into physical space that are energetically favorable while maintaining topologies that support efficiency and robustness. For example, it is thought that across technological [32], neural [39, 40], and distribution [54, 55] networks alike, one such common feature is hierarchical structure. To begin investigating these questions here, we next assessed each network for the presence of topological and physical Rentian scaling, following the methods described in Rentian scaling.

**Fig 5:**
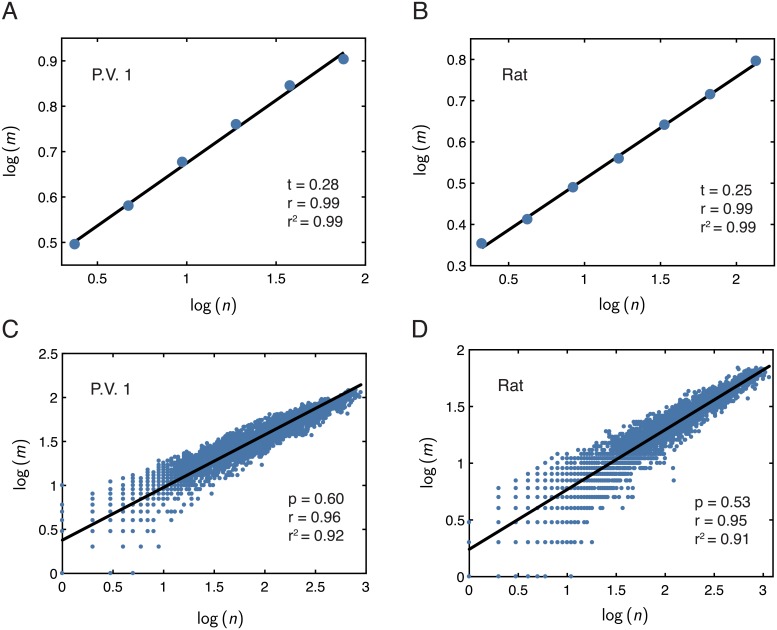
Rentian scaling analysis of the distribution networks. *(A,B)* Examples of log *m* *vs.* log *n* computed from a topological partitioning of a network from the mycelium *P.V. 1* and from a topological partitioning of an arterial network from a rat brain, respectively. *(C,D)* Examples of log *m* *vs.* log *n* computed from a physical partitioning of a network from the mycelium *P.V. 1* and from a physical partitioning of an arterial network from a rat brain, respectively. In panels *(A–D)* the black lines correspond to a simple linear regression, from which we estimate the displayed scaling exponents *t* or *p*, describing either the topological or physical Rentian scaling relationships, respectively. The *r*-value is the Pearson correlation coefficient and the *r*^2^-value is the coefficient of determination. Additional examples of these relationships for other networks are shown in Fig. F and Fig. H in the S1 Text.

We first computed topological Rentian scaling. Fig 5A and 5B show examples of log *m* *vs.* log *n* obtained from a min-cut bi-partitioning method applied to a rat vasculature network and a mycelial network, respectively. (Fig. F in the S1 Text shows examples from the other distribution networks as well). Visual inspection suggested that *m* and *n* scale with one another in log-log space, permitting the estimation of a topological Rent exponent *t*. We also calculated the linear correlation coefficient and a linear regression between log *m* and log *n* (depicted as the black lines through the data in the examples of Fig 5A and 5B) for each distribution network. When averaged over several runs of the topological partitioning process, we found *r* ≥ 0.991 and *r*^2^ > 0.981 for all networks, providing further evidence of Rentian scaling in topological space. (See S1 Text for further details regarding this analysis).

We turn next to physical Rentian scaling. In the context of the distribution networks, this analysis evaluates whether the spatial layout of a network is such that the number *m* of vessels or cords passing into or out of a given contiguous region of a network scales with the number of nodes *n* inside that region, and whether such a relation is conserved over different length scales and spatial domains of a network. Fig 5C and 5D show examples of log *m* *vs.* log *n* in a mycelial system and a vasculature system, respectively, and Fig. H in the S1 Text shows examples from each of the other kinds of distribution networks as well. Visual observation provided initial evidence for the existence of physical Rentian scaling in the distribution networks, and to further assess each network for this relationship, we also computed a linear correlation and a linear-regression between log *m* *vs.* log *n* (displayed as the black lines over the data in Fig 5C and 5D). For all networks, we found Pearson’s correlation coefficients of *r* > 0.9 and coefficients of determination *r*^2^ > 0.82, which further indicated that the functional form *m* ∝ *n*^*p*^ was a relatively good fit to the data and that we could estimate physical Rentian scaling exponents *p* for each network from the linear-regression. (See S1 Text for further details on this analysis). The presence of physical Rentian scaling suggests that the connectivity of the distribution networks exhibits a type of hierarchical organization and space-filling capacity in terms of their physical arrangement and embedding into real space.

**Fig 6:**
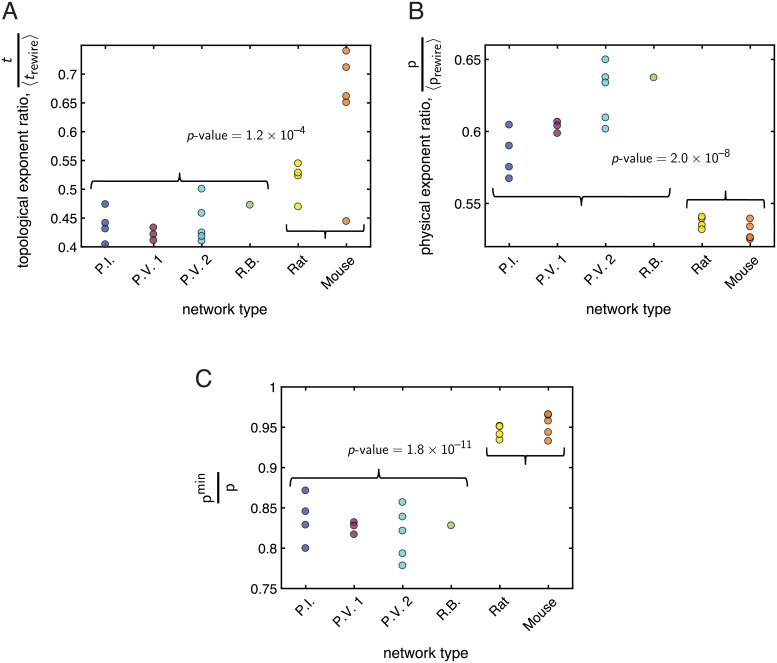
Comparison of Rentian scaling analysis in the mycelial networks and vasculature networks. In each panel, the *x*-axis labels the kind of network, with the curly braces grouping the mycelia (first 4 networks) and the vasculature (last 2 networks). The *y*-axis measures the value of a given property for each network. A *p*-value is displayed if there was a statistically significant difference (as determined by a two-sample *t*-test) in the mean value of the given property between the population of mycelial networks and the population of vasculature networks. *(A)* The normalized topological Rent exponent $\tilde{t}=t/\langle t_{\text{rewire}}\rangle$ was significantly smaller in the population of mycelial networks compared to the population of vasculature networks. *(B)* The normalized physical Rent exponent $\tilde{p}=p/\langle p_{\text{rewire}}\rangle$ was significantly larger in the population of mycelial networks compared to the population of vasculature networks. *(C)* The ratio *p*^min^/*p* of the theoretical minimum physical Rent exponent to the true physical Rent exponent was signficantly smaller in the population of mycelial networks compared to the population of vasculature networks. See the main text for more details on each measure.

Though we can estimate physical and topological Rent exponents for the empirical networks from best-fit lines to log *m* *vs.* log *n*, it is important to note that because the networks considered here are relatively small, the values of the exponents should be considered carefully, as should a direct comparison across different networks. In order to give context and meaning to the exponent values, we thus compared the scaling relationships from the empirical networks to those of their randomly rewired benchmarks (see Null models). Keeping the node locations for the randomly-rewired null models the same as the corresponding empirical network, we considered the ratios $\tilde{t}=t/\langle t_{\text{rewire}}\rangle$ and $\tilde{p}=p/\langle p_{\text{rewire}}\rangle$. Comparing $\tilde{t}$ between the vasculature and mycelial systems (Fig 6A) revealed a statistically significant distinction between the two kinds of networks (*p*-value = 1.2 × 10^−4^). In particular, the population average of the topological Rent exponent ratio was smaller for the set of mycelial networks compared to the set of vasculature networks ($\bar{{\tilde{t}}_{F}}=0.44$, $\bar{{\tilde{t}}_{V}}=0.59$), suggesting that in terms of topological complexity, the vasculature networks are on average more similar to their randomly-rewired benchmarks. We also found a clear relative difference in the physical Rent exponent ratios of the vasculature *vs.* fungal networks (Fig 6B), with the population of vasculature networks having smaller mean physical Rent exponent ratio ($\bar{{\tilde{p}}_{V}}=0.53$, $\bar{{\tilde{p}}_{F}}=0.61$). The difference between the two groups was statistically significant (*p*-value = 2.0 × 10^−8^), and indicates that, compared to the mycelial networks, the physical Rent exponents of the vasculature networks are even smaller than the mean exponent from their ensemble of random null models.

Because we could estimate physical and topological Rent exponents for each empirical network and their randomly rewired null models, we also examined how the physical Rent exponent of each network compared to its theoretical minimum value (see Rentian scaling). We first considered the quantity $p^{\text{min}}/p-\langle p_{\text{rewire}}^{\text{min}}/p_{\text{rewire}}\rangle$ for each network, where *p* is the true physical Rent exponent of an empirical network and *p*^min^ is its theoretical minimum value, and where *p*_rewire_ is the true physical Rent exponent of a corresponding randomly rewired benchmark network and $p_{\text{rewire}}^{\text{min}}$ is its theoretical minimum value; the angled brackets indicate an average over the ensemble of null-model networks. As perhaps expected, we found $p^{\text{min}}/p-\langle p_{\text{rewire}}^{\text{min}}/p_{\text{rewire}}\rangle >0$ for all networks. Thus, the empirical distribution networks have physical Rent exponents closer to their minimum possible values, indicating that they achieve more efficient spatial layouts relative to their set of randomly-rewired null-models. We next compared the quantity *p*^min^/*p* across the vasculature and mycelial networks (Fig 6C), finding a statistically significant difference between the two groups ($\bar{p_{\text{F}}^{\text{min}}/p_{\text{F}}}=0.83$, $\bar{p_{\text{V}}^{\text{min}}/p_{\text{V}}}=0.95$, *p*-value = 1.8 × 10^−11^). The vasculature networks yielded values of *p*^min^/*p* closer to one (i.e., had physical Rent exponents closer to their theoretical minimum) compared to the fungal networks, suggesting that they may be especially efficiently embedded. We explore this idea further in the next section. Also see the S1 Text for an analysis of topological and physical Rentian scaling in simulated networks developed from biologically inspired optimization principles [53], in which we also find evidence of these relationships as well as exponent values similar to those obtained from the empirical data.

### Comparative analysis of network organization using biologically-motivated measures

While there are some general similarities between the vasculature and mycelial networks, differing habitats or environmental pressures could lead to distinct structural features. Indeed, we quantified some of these distinctions in the previous section, Characterization of network architecture with graph-theoretical measures. Here we conduct further network-based analyses that more explicitly take into account the spatial embedding of these systems, and we compare the vasculature and fungi using a set of network properties motivated specifically by the functional requirements of biological distribution networks.

#### Tradeoffs between physical network efficiency and wiring

We begin by examining the physical efficiency *E*^p^ and the wiring length *W* of each network. The physical efficiency (Eq 3) is a measure that reflects the routing capabilities of a network [56], and importantly, because *E*^p^ is defined from the Euclidean lengths of network edges—rather than topological distances—it takes into account the spatial layout of a system. Intuitively, higher efficiencies may allow for improved transport of fluid and nutrients throughout a network, which are desirable capabilities for both vasculature and mycelial systems. In general, it is expected that increases in the number of connections between the same set of nodes will improve network efficiency by creating shortcuts between pairs of otherwise more distant regions. But, an addition of edges will in turn increase the amount of material used to construct the network. Note that here we estimate the material cost—or more precisely, the wiring length—to be the sum of the physical lengths of all edges in the system (Eq 2). As noted previously and in the discussion section below, an improved quantification of network cost would also include radial information as well, but this data was unavailable for the vasculature systems.

In order to quantify the extent to which each network was organized for low wiring length or high physical efficiency—and to compare and contrast the balance between these competing network features across the two types of distribution networks—we considered the relative quantities *W*_rel_ (Eq 5) and $E_{\text{rel}}^{p}$ (Eq 6). These measures reflect how close a given network is to two spatially-informed null models [44–46], the minimum spanning tree *MST* and the greedy triangulation *GT*. Given a set of node locations, the *MST* represents a low cost but also low efficiency planar graph, and the *GT* represents a high cost but also high efficiency planar graph [44]. Recall that $E_{\text{rel}}^{p}$ and *W*_rel_ are bounded between 0 and 1 to reflect the similarity of the empirical networks to their *MST* (where the relative measures are equal to zero) and the *GT* (where the relative measures are equal to one), respectively (see Null models for more details on the null models and relative measures).

**Fig 7:**
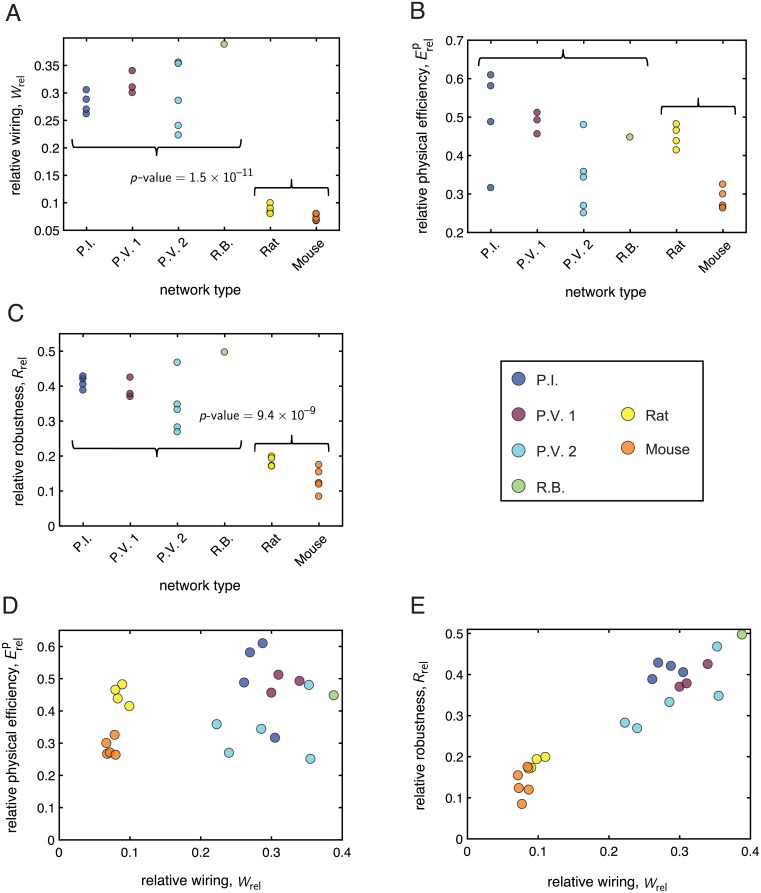
Mycelial and vasculature networks exhibit distinct tradeoffs between wiring, physical efficiency, and structural robustness. In panels *(A-C)* the *x*-axis labels the kind of network, with the curly braces grouping the mycelia (first 4 networks) and the vasculature (last 2 networks). The *y*-axis measures the value of a given property for each network. A *p*-value is displayed if there was a statistically significant difference (as determined by a two-sample *t*-test) in the mean value of the given property between the population of mycelial networks and the population of vasculature networks. *(A)* The mean relative wiring for all networks was significantly larger for the population of mycelial networks compared to the population of vasculature networks. *(B)* The difference in the mean relative physical efficiency of the mycelial networks and the vasculature networks was not statistically significant. *(C)* The mean relative structural robustness was significantly larger for the population of mycelial networks compared to the population of vasculature networks. *(D)* A scatterplot of the relative physical efficiency *vs.* the relative wiring for each network. *(E)* A scatterplot of the relative structural robustness *vs.* the relative wiring for each network. For all plots, the relative quantities *W*_rel_, $E_{\text{rel}}^{p}$, and *R*_rel_ were determined by normalizing each network with respect to its own spatial null models, which then allowed for a meaningful comparison of the different types of distribution networks to one another.

The relative wiring length *W*_rel_ (Fig 7A) satisfied *W*_rel_ < 0.4 for all networks, suggesting the presence of general constraints on the structure and wiring usage in these systems. Grouping together all mycelial networks into one set and all vasculature networks into another set, we used a two-sample *t*-test to further determine whether there were statistical differences in the mean of *W*_rel_ between the two populations. We found that the mean relative wiring was indeed significantly different between the two groups (*p*-value = 1.5 × 10^−11^), with the population of vasculature networks having a lower mean relative wiring compared to that of the population of mycelial networks ($\bar{W_{\text{rel},V}}=0.08$, $\bar{W_{\text{rel},F}}=0.30$). We next examined the relative physical efficiency $E_{\text{rel}}^{p}$, which was intermediately valued for all networks (Fig 7B), ranging between 0.25 and 0.61. Interestingly, we found that the difference in the mean relative efficiency measure was not statistically significant across the two groups ($\bar{E_{\text{rel},F}^{p}}=0.43$, $\bar{E_{\text{rel},V}^{p}}=0.36$, *p*-value >0.05). Thus, while both sets of networks appear more similarly optimized for physical efficiency, our results indicate that the wiring constraints are stronger for the vasculature networks than for the fungi. This suggests that there may be different kinds of pressures driving the network organization of the two types of distribution systems.

To further investigate the tradeoffs between the relative efficiency and relative wiring, we plotted $E_{\text{rel}}^{p}$ *vs.* *W*_rel_ (Fig 7D). We observed that the vasculature consistently satisfied $W_{\text{rel}}<E_{\text{rel}}^{\text{p}}$, with a significant margin between the two quantities. A similar finding held for several of the fungi, but we also found that some of the mycelial networks sat just above, on, or sometimes below the line of equality ($W_{\text{rel}}=E_{\text{rel}}^{p}$). This result implies that for a given wiring, the vasculature networks can achieve a relatively greater physical efficiency—suggesting an economical, well-organized architecture—compared to several of the fungal networks, which appeared to have a less-optimal use of extra wiring, as measured by the ability of that wiring to increase the relative efficiency of the network. Moreover, for significantly lower *W*_rel_, the rat vasculature achieved similar or higher $E_{\text{rel}}^{p}$ values than many of the fungi, indicating an especially beneficial use of material in the vasculature systems. Finally, we note that the rodent data was much more tightly clustered in this phase space compared to the more varied fungal data; this points to consistent, regularized network structure in the vasculature in contrast to more diversity in the mycelial networks.

#### Differences in network robustness

To better understand the implications of the wiring-efficiency tradeoff, we also considered the structural robustness of each network, which—like efficiency—should be facilitated by increased wiring. Robustness is an important consideration in both mycelial and vasculature networks. In mycelia, damage can occur from factors such as rough environmental conditions or from predation [17, 29–31], and in vasculature, a common source of damage is from the formation of blood clots that block vessels and prevent flow, leading to stroke. Previous work on fungal networks has shown that these organisms can achieve high levels of robustness to attack [17, 21]. In a study on rodent vasculature [27], interconnected loops were found to provide robustness to the backbone of surface vessels under edge deletion, and after occlusion to a surface arteriole, the backbone was able to re-route blood flow and preserve the surrounding neuronal tissue.

Here, we let the robustness *R* of a network be the percentage of edges removed in order for the size of the largest connected component to drop to half of its original value [44, 45], and we considered the *relative robustness* *R*_rel_ (see Biophysically-motivated network measures for details) for comparison of the fungi and vasculature. Fig 7C shows the relative robustness for all networks, which ranged between 0.08 and 0.50. Using a two-sample *t*-test to compare *R*_rel_ between the population of fungal networks and the population of vasculature networks, we found that the means of the two groups were significantly different ($\bar{R_{\text{rel},F}}=0.38$, $\bar{R_{\text{rel},V}}=0.15$; *p*-value = 9.4 × 10^−9^). This clear separation between the mycelial networks and the vasculature networks suggests that the mycelia may be better optimized for resistance to damage, and further points to the fact that although comparable in certain ways, the two types of transport networks indeed exhibit different structural organization and tradeoffs. We also observed a positive trend between *R*_rel_ and *W*_rel_ across all networks (Fig 7E), indicating that increasing the amount of wiring used to connect a given set of nodes strongly correlates with improved resistance to network damage. (As might be expected, we additionally found a positive trend between *R*_rel_ and a relative measure of network density, where instead of using the total wiring length in Eq 5, we used the total number of edges in each network). There was not such a strong relationship between $E_{\text{rel}}^{p}$ and *W*_rel_, suggesting that while increased connectivity may partially improve network efficiency, the specific placement of additional wiring in the network can play a role as well.

**Fig 8:**
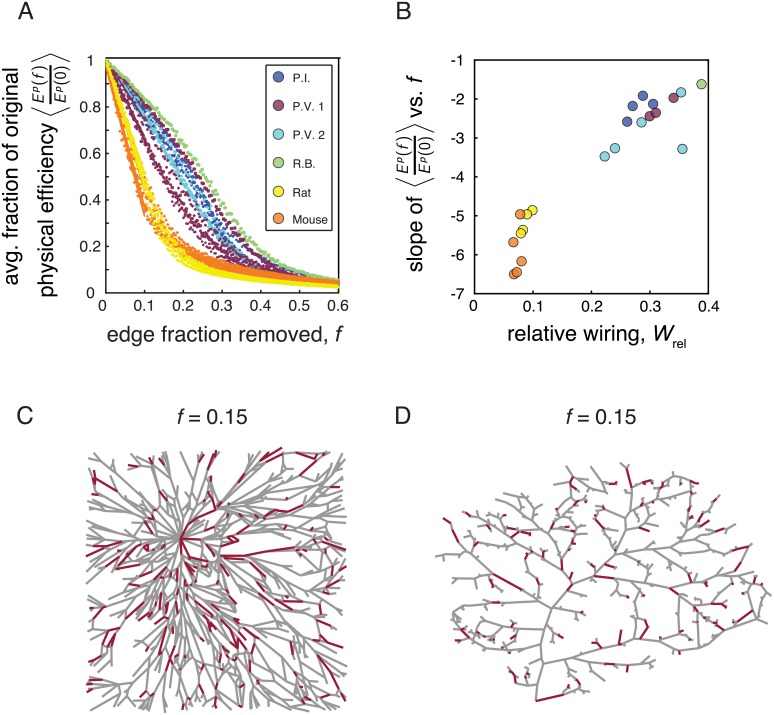
Decline of physical efficiency with random edge removal. *(A)* Ensemble average of the ratio of the physical efficiency of damaged networks to the physical efficiency of the original network, as a function of the edge fraction removed. The efficiency of the vasculature networks declined more rapidly than that of the fungal networks. *(B)* The drop-off in physical efficiency was approximated for each network as the slope of a linear fit to *E*^p^(*f*)/*E*^p^(0) *vs.* *f*, computed between *f* = 0 and *f* = 0.1. There was a positive correlation between the slope and the relative wiring of the networks. *(C)* An example of a network from *P.V. 2*, where the red lines correspond to a random selection of *f* = 0.15 of the total number of edges, and the gray lines correspond to the remaining edges in the network. *(D)* An example of a network of a mouse vasculature system, where the red lines correspond to a random selection of *f* = 0.15 of the total number of edges, and the gray lines correspond to the remaining edges in the network.

Given that both the vasculature and fungal systems are subject to certain forms of impairment, another important question is how the physical efficiency of the underlying networks changes as a result of random damage. In other words, how functional does the topology of these networks remain when edges are removed? In order to examine this question, we tracked the change in the global physical efficiency of each network as a function of edge fraction removed. More specifically, if we let *f* represent the fraction of edges removed, we considered a range *f* = 0 to *f* = 0.6, in steps of Δ*f* = 0.004. At each step, we removed the corresponding fraction of edges at random from each network, and computed the global physical efficiency (Eq 3) *E*^p^(*f*) of the perturbed system and also the ratio *E*^p^(*f*)/*E*^p^(0). This process was repeated 20 times for each fraction *f*, and we considered the ensemble average 〈*E*^p^(*f*)/*E*^p^(0) 〉 over the 20 trials. The structure of the resulting curves of 〈*E*^p^(*f*)/*E*^p^(0)〉 *vs.* *f* (see Fig 8A) provides insight into how the transport capabilities of each network are altered under random damage.

We observed that the efficiency of the vasculature networks falls off more rapidly than the efficiency of any of the mycelial networks (Fig 8A). In order to help visualize the effect of the edge removal, we show an example of a robust fungal network (Fig 8C) and more delicate vasculature network (Fig 8D); in each case, the red lines correspond to a fraction *f* = 0.15 of edges selected at random. To quantify the steepness of the decline in physical efficiency for realistic removal fractions, we considered the slope of linear fits to 〈*E*^p^(*f*)/*E*^p^(0)〉 *vs.* *f* between *f* = 0 and *f* = 0.1. Then, we asked whether the steepness of the fall-off (estimated by the slope of the linear fit to each curve) was related to the relative wiring of the network. We found that *W*_rel_ was indeed a good predictor of the decay in physical efficiency (Pearson correlation *r* = 0.95, *p*-value = 2.85 × 10^−11^; Fig 8B). This analysis highlights the fragility of the vasculature systems, whose ability to maintain their transport efficiency is impaired quickly with damage compared to the fungal networks, which appear better able to maintain this function in the face of edge loss.

## Discussion

In this study, we examined the structural networks formed by two classes of biological distribution systems: lab-grown mycelial networks and vasculature networks extracted from the surface of rodent brains. While each of these networks have been analyzed independently, these systems also have different environmental conditions, growth processes, and transport mechanisms, which may differentially impact their network organization. Thus, our goal here was to quantitatively compare and contrast the mycelia and vasculature using methods from graph theory and network science, in order to gain insight into possible variabilities that may exist across the two systems. Though the two types of distribution networks share common organizational constraints—such as two-dimensional planar topologies—we indeed uncovered a number of distinctions in the architectures of the two families of biological transport networks.

The first part of our investigation focused primarily on characterizing the structure of the fungi and vasculature networks with a collection of classic network-theoretic measures that assess different kinds of organization across a range of scales, from local metrics such as degree, to diagnostics that probe paths of connectivity globally throughout a network. When necessary, we controlled for differences in network size and density by normalizing measures to their values in an ensemble of randomly rewired null models that preserved both the size and degree distribution of each empirical network. Our analysis revealed statistically significant differences in topological properties including mean degree, clustering coefficient, and alpha index—indicating variability of local structure and loop density between the mycelial and vasculature networks. A crucial feature of the distribution networks studied here is that they are also embedded into real space, and thus Euclidean distances and the spatial layout of the networks are important considerations. While many network measures are agnostic to the spatial organization of nodes and edges, there are some measures—such as path efficiency and edge betweenness centrality—that have both topological (i.e. based only on connectivity information) and physical (i.e. also incorporate spatial information) counterparts. One interesting result was that the vasculature tended to exhibit a greater degree of similarity between topological and physical edge betweenness centrality as compared to the fungal networks, a finding that motivated the more physically and biologically informed network analysis carried out in the latter portion of this study.

We also analyzed the mycelial and vasculature networks for Rentian scaling, which is a kind of hierarchical structure that can exist in both the physical and topological space of a network. This property has previously been found in the London Underground transportation network [3] and in biological neural systems [39, 40], but has yet to be examined in biological transport networks. It has been suggested that biological distribution networks, including blood vasculature, respiratory systems, and mycelia, are fractal-like and space filling [54, 57, 58], and these properties have been proposed to validate and explain scaling laws that relate metabolic rates to body mass [59]. More recently, hierarchical organization in the form of nested loops has been studied in leaf venation [55], and this structure has been shown to arise in networks optimized for resistance to damage and fluctuations in load [53, 60, 61]. Providing quantitative measures of what it means to have hierarchical organization is thus an important line of inquiry for spatially embedded and biological networks. We found that common design features of both the mycelial and vasculature networks were physical and topological Rentian scaling, which imply conserved relationships across length scales between the number of nodes in a given region of the network, and the number of cords or vessels crossing the region boundary. We also found evidence of Rentian scaling in simulated networks optimized for transport efficiency in the presence of fluctuations and constraints on network “cost”, providing some insight into how such structure might arise. Importantly, without the space-filling nature that arises in the model networks, one could still uncover topological scaling, but large spatial inhomogeneities in either the distribution of nodes or edges would destroy the scaling in real space. Computing ratios of the empirical Rent exponents to their values in randomly-rewired size and density preserving benchmarks, we found that the vasculature exhibited lower physical Rent exponent ratios, but on average larger topological Rent exponent ratios. These results suggest that the vasculature have particularly constrained layouts, but maintain relatively high topological complexity, given their size and degree distribution. Moreover, we found that the vasculature networks achieved physical Rent exponents closer to their theoretical minimum values, further indicative of an especially efficient placement of network components into real space.

In the second part of this study, we carried out a network-based analysis that more purposefully considered the spatial embedding of the distribution systems, and we examined network properties more directly motivated by the structural and functional requirements of these systems. Specifically, we examined three desirable but often competing characteristics—wiring costs, transport efficiency, and structural robustness—finding some clear constrasts in how the vasculature and fungi balance network-based measures of these quantities. In particular, though both kinds of networks had comparable physical efficiencies, the vasculature had lower wiring length and robustness than the mycelial networks. One hypothesis is that the differential tradeoffs observed in mycelial *vs.* vasculature networks may reflect their differing functional requirements and environmental settings. To be effective, the vasculature networks must supply a fixed area with blood, and thus experience a notion of boundary conditions and minimum requirements that may constrain the possibilities or necessity for further growth. In contrast, the growth of mycelial networks is not constrained by a pre-allocated or bounded region of space that they must supply. One hypothesis for the more minimal wiring in the vasculature may be that it is energetically necessary and that more cost-efficient networks are sustainable due to the confined and protected setting of these networks. Perhaps the vasculature can afford to devote less material to the network because it resides in a regulated and highly controlled setting, whereas the mycelial systems typically grow under more heterogeneous environmental conditions.

In particular, environmental changes and patchy habitats (e.g., the soil and availability of water and nutrients) are likely to play a large role in the supply of nutrients to the fungi and can impact network development [62]. On the other hand, the supply of oxygenated blood through the carotids to the brain is well regulated and near constant; thus, we might expect less load variation in the vasculature compared to the fungal systems. Importantly, these differences could lead to the observed variability of the networks’ robustness and relationships between wiring length and efficiency. The vasculature systems are able to achieve relatively high efficiency with low wiring, suggesting standardized network architectures that are structured in order to utilize a small amount of wiring redundancy in a way that significantly improves the capabilities of the network to route resources. In contrast, the mycelial networks strike a different balance, having larger wiring lengths and not always consistent organization such that higher relative wiring directly yields relatively greater efficiencies. This indicates less standardized and perhaps less optimized network architectures. Notably, however, the tradeoff of greater material cost in the mycelial systems directly yields improved resistance to damage, and the ability to maintain higher functionality under perturbation of the network. This feature may be demanded for survival or driven by the more varied and unprotected environmental conditions in which these systems reside and have evolved.

### Methodological considerations and future directions

There are a number of methodological considerations—especially concerning the construction of the network representations of the vasculature and fungi—that are useful to comment on. First, it is crucial to point out that we have used a simplified network representation of the distribution systems in that information about cord or vessel radii was neglected. The main reason for this was that these details were unavailable for the vasculature. However, it is known that tube area is important functionally, and can significantly alter transport in distribution networks since it affects flow resistance. Including this additional physical property would thus yield a more complete analysis, both in terms of quantifying network construction costs as well as measuring network properties such as physical efficiency. Indeed, past studies on mycelial networks have found that the presence of radial thicknesses confers improved transport characteristics to the system [16, 18, 20]. It would be interesting to take this into account in future work to investigate whether the inclusion of radial information in the weighting of the network reduces or enhances the differences found between the vasculature and mycelial systems. It is also important to point out that the definition of wiring length employed in this study assumes a linear relationship between edge length and edge “cost”. In reality this might not be the case. For example, longer connections might be disproportionately more expensive to build and maintain, or there might be an offset cost to create connections at all. Furthermore, we note that the network property of physical efficiency quantifies transport capabilities only in terms of shortest paths, and in the case studied here, assumes bi-directionality of flow; an understanding of transport along indirect walks is also relevant in distribution systems, as is the notion of directed transport that allows for the movement of nutrients through long distances. The extension of traditional network measures and null models to include this type of information may lead to considerable insight into structure-function relationships in biological transport networks more generally. For example, network communicability [63] naturally includes information on walks of all lengths between pairs of nodes, and could be one valuable candidate for further analyses. In addition, it is worth noting that the completeness of the network representations are also subject to the resolution at which fine-scale edges can be traced or digitized.

We also note that in the network representation of the mycelial networks, the food source itself was considered as an imposed ‘super-node’ that connects to the cords incident on its boundary. Since the mycelial networks grow outward from the inocula, the density of edges around that node may be higher compared to more peripheral regions of the network, which could introduce some heterogeneity in the architecture. In terms of Rentian scaling in particular, we would then expect partitions that surround the ‘super-node’ to have a higher number of boundary-crossing edges. These partitions might be a cause for some spread or disruption in the spatial scaling relationship, or perhaps bias the exponent towards higher values. However, at least upon visual inspection of the examples of log *m* *vs.* log *n* in Fig 5 and Fig. H in the S1 Text, this does not appear to be a salient effect. Since we sample the network numerous times to compute the scaling, most of the partitions will not include the inocula, and this likely prevents the ‘super-node’ from dominating the scaling. However, it is important to be aware of the issues that the ‘core-like’ structure in the network can induce.

In regard to the vasculature networks, it is important to remark on the fact that nodes correspond to either branching points among surface vessels or places where penetrating arterioles descend into the underlying tissue. Furthermore, many of the penetrating arterioles originate from offshoots that stem from the network of surface vessels and that terminate at the end of a surface edge in a single point [27]. Though this is a meaningful organizational feature of the vasculature that we chose to include in our primary examination, it is still interesting to consider whether results hold if we consider a “reduced” network that does not contain the stubs leading to single penetrating arterioles. To this end, we carried out an analysis comparing the complete vasculature networks to reduced versions in which isolated penetrating arterioles (and the corresponding surface edges connecting to them) were removed, and we also compared the reduced vasculature networks to the mycelial networks in order to test the robustness of the main results of this study (full details are provided in the S1 Text). In summary, we found that though a number of network properties differ between the full and reduced vasculature networks, these differences are slight and do not change the conclusions or significance of the main results in terms of how the organization of the vasculature networks compare to that of the mycelial networks.

Aside from those already mentioned, there are several other directions for future work. For example, one could try to further understand the observed differences between the structure of the vasculature and fungi, but perhaps more importantly, investigate the causes and actual functional consequences of those differences. We suggested that some of the observed variabilities in network organization may be due to the different environmental habitats or function of the two kinds of transport systems. However, to more solidly establish this will certainly require both further experimental work, as well as more extensive theoretical models. For example, it would be interesting to build off of prior work [9, 14, 16, 19, 20, 25, 27, 29–31, 55, 61, 64] and continue to design experiments and construct models that describe how a distribution network evolves in a physically unbounded and dynamically variable environment, *vs.* in a spatially constrained and more regulated environment, and investigate how these differences affect network adaptation and the resulting network architecture. Furthermore, while this work considers only the static network structure, an improved analysis should also consider the relation of that structure to experimental data on flow throughout the network, or should investigate the functional consequences of certain structural network properties using models of flow and transport. Understanding how transport in turn affects network development is also important [16, 61]. Another intriguing direction would be to further examine the properties of distribution networks that are embedded not in 2-dimensions, but in 3-dimensions [26, 65], where relationships between topology and geometric structure or spatial layout may be more complex.

### Conclusion

In this study, we used network-based methods to quantitatively compare and contrast the structural skeleton of mycelial networks and vasculature networks from the surface of rodent brains. Both systems are two-dimensional, planar, biological transport networks, whose organization arises through natural processes in the absence of global rules for network construction. However, despite similarities, these networks have different developmental and transport mechanisms, and exist in distinct environments. While such differences are likely to differentially impact the network structure of vasculature and mycelia, little work has focused on comparative analyses of network traits across the two systems. Here we characterized the vasculature and mycelial networks using both classic network metrics, as well as a collection of measures inspired by the functional requirements and physical constraints of and on these systems. We quantified and described both conserved features of network organization across the two types of transport networks, as well as differences in topological structure and in tradeoffs between network correlates of wiring length, efficiency, and structural robustness. This work illustrates the utility of network science to uncover common organizational properties across different kinds of networks, as well as variabilities that may be important to or reflect differences in the evolution, functional requirements, or capabilities of different systems.

## Supporting information

S1 Text Additional figures, further description of methods, and supplementary analyses. The supplementary text includes figures showing examples of the different kinds of mycelial networks and vasculature networks examined in this study. It also provides more in-depth descriptions and formal definitions of the standard topological graph metrics utilized in this study, further details on the modeling and simulations of the optimal transport networks, additional information regarding the physical and topological Rentian scaling analysis, a supplementary examination of structural network robustness, and a more in-depth description and investigation of the reduced versions of the vasculature networks in which penetrating arteriole offshoots are discarded.
(PDF)
