# Supplementary material for: Comparing two classes of biological distribution systems using network analysis
Source: PLoS Comput Biol. 2018 Sep 7;14(9):e1006428. doi: 10.1371/journal.pcbi.1006428 (PMC6145589; doi:10.1371/journal.pcbi.1006428)
Supplement: S1 Text — The supplementary text includes figures showing examples of the different kinds of mycelial networks and vasculature networks examined in this study. It also provides more in-depth descriptions and formal definitions of the standard topological graph metrics utilized in this study, further details on the modeling and simulations of the optimal transport networks, additional information regarding the physical and topological Rentian scaling analysis, a supplementary examination of structural network robustness, and a more in-depth description and investigation of the reduced versions of the vasculature networks in which penetrating arteriole offshoots are discarded. (PDF) [file pcbi.1006428.s001.pdf]

Supplementary Text

"Comparing two classes of biological distribution systems using network analysis"

Lia Papadopoulos[1], Pablo Blinder[2,3], Henrik Ronellenfitsch[1,4], Florian Klimm[5,6], Eleni Katifori[1], David Kleinfeld[7], Danielle S. Bassett[1,8,9,10*],

1 Department of Physics & Astronomy, University of Pennsylvania, Philadelphia, Pennsylvania, United States of America
2 Sagol School of Neuroscience, Tel-Aviv University, Tel Aviv, Israel
3 Department of Neurobiology, George S. Wise Faculty of Life Sciences, Tel Aviv University, Tel Aviv, Israel
4 Department of Mathematics, Massachusetts Institute of Technology, Cambridge, Massachusetts, United States of America
5 Mathematical Institute, University of Oxford, Oxford, United Kingdom
6 Systems Approaches to Biomedical Science Doctoral Training Centre, University of Oxford, Oxford, United Kingdom
7 Department of Physics, University of California San Diego, La Jolla, California, United States of America
8 Section of Neurobiology, University of California San Diego, La Jolla, California, United States of America
9 Department of Bioengineering, University of Pennsylvania, Philadelphia, Pennsylvania, United States of America
10 Department of Electrical & Systems Engineering, University of Pennsylvania, Philadelphia, Pennsylvania, United States of America
11 Department of Neurology, University of Pennsylvania, Philadelphia, Pennsylvania, United States of America

* dsb@seas.upenn.edu

Additional figures of network data

Mycelial networks

We showed an example of the network formed by *Phallus impudicus* (*P.I.*) in Fig. 1A of the main text. Here in Fig. A(i)–(iii), we also provide examples of *Phanerochaete velutina* grown from five inocula (*P.V. 1*), *Phanerochaete velutina* grown from a single inocula (*P.V. 2*), and *Resinicium bicolor* (*R.B.*), respectively.

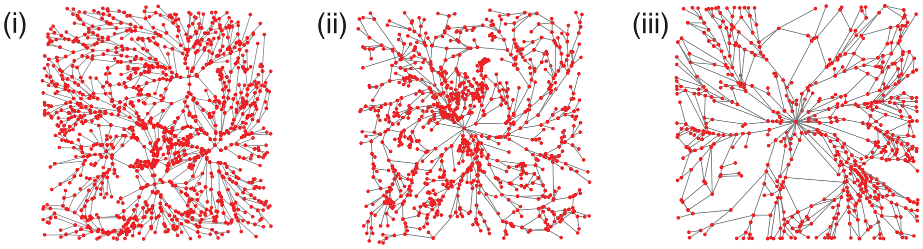

Fig A. Additional examples of the mycelial networks. *(i)* The network structure formed by *Phanerochaete velutina* grown from five inocula (*P.V. 1*). *(ii)* The network structure formed by *Phanerochaete velutina* grown from a single inoculum (*P.V. 2*). *(iii)* The network structure formed by *Resinicium bicolor* (*R.B.*) grown from a single inoculum.

Rodent vasculature networks

An example of a vasculature network from a rat brain was shown in Fig. 1B of the main text; here (Fig. B) we also include an example vasculature network from a mouse brain. Red nodes correspond to branching points among surface vessels, and blue nodes are penetrating arterioles.

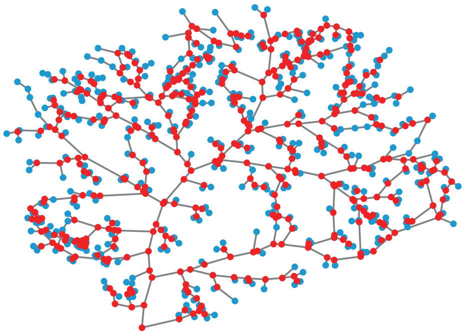

Fig B. An example of a vasculature network from mouse neocortex. This figure shows the entire pial network from the territory of the middle cerebral artery in a mouse brain. Nodes are either branching points (shown in red) or penetrating arterioles (shown in blue), and edges are vessel segments (depicted as gray lines).

Detailed descriptions of standard topological graph metrics

In this section we provide more in-depth definitions of the classic topological network measures analyzed in the main text. These include the degree, clustering coefficient, alpha index, topological efficiency, and topological edge-betweenness centrality.

Degree

The *degree* k_i of a node i is the number of edges incident on that node, and is a local measure of network connectivity. The mean degree $\langle k \rangle$ of a network is defined as the mean of k_i over all nodes in the network.

Clustering coefficient

The *clustering coefficient* C_i of a node i can be calculated as

$$C_i = \sum_{hj} \frac{A_{hj} A_{ih} A_{ij}}{k_i(k_i - 1)}, \tag{1}$$

and gives the number of fully-connected 3-node subgraphs (i.e. triangles) involving node i, divided by the number of triples centered at node i [1]. The mean clustering coefficient C of the network is defined as the mean of C_i over all nodes in the network.

Alpha index

Another useful measure for planar networks is the *alpha index* (or *"meshedness"*), α. This topological quantity gives the total number of elementary cycles

in a planar network, divided by the largest possible number of elementary cycles that can exist in a planar network with the same number of nodes [2]. In terms of the number of nodes N and number of edges M in the network, the alpha index is defined as

$$\alpha = \frac{M - N + 1}{2N - 5}. \tag{2}$$

While the clustering coefficient considers only the presence of cycles of length three, the alpha index takes into account elementary cycles of all lengths (where the length of a cycle is equal to the number of edges that compose it).

Topological efficiency

Important concepts in network analysis are those of paths and path lengths. A path through a network is a non-intersecting sequence of consecutive edges (i.e. edges that share a common node). The topological path length is then given by the total number of edges in the path. It is often useful to consider the shortest paths in a network, which are those paths that minimize the total number of "hops" or edges that must be traversed in order to move between a given pair of nodes. Knowing the shortest topological paths (or "hop" distances) between all node pairs allows for the calculation of the average *topological efficiency* [3], $E^{\mathrm{t}}_{\mathrm{avg}}$, which has been proposed as one way to quantify the ease of information exchange or transport between nodes of a network. Denoting the length of the shortest topological path between i and j as l^{t}_{ij}, the average efficiency is given by

$$E^{\mathrm{t}}_{\mathrm{avg}} = \frac{1}{N(N-1)} \sum_{i,j} \frac{1}{l^{\mathrm{t}}_{ij}}. \tag{3}$$

It is common practice to normalize the topological efficiency for a given network with N nodes by its value for a fully-connected N-node network. This results in the *global topological efficiency* E^{t}, where $0 \leq E^{\mathrm{t}} \leq 1$.

Topological edge betweenness centrality

The *topological edge betweenness centrality* B^{t}_e is another measure based on shortest paths, and is one way to quantify the importance of a particular edge in a network. For a given edge (i,j), it is defined as the fraction of shortest paths between all pairs of nodes that pass through that edge. In particular, we calculate

$$B^{\mathrm{t}}_e(i,j) = \sum_{q,r} \frac{\psi^{\mathrm{t}}_{qr}(i,j)}{\psi^{\mathrm{t}}_{qr}}, \tag{4}$$

where $\frac{\psi^{\mathrm{t}}_{qr}(i,j)}{\psi^{\mathrm{t}}_{qr}}$ is the fraction of shortest paths (measured in topological units of distance, i.e. number of edges) between q and r that traverse edge (i,j).

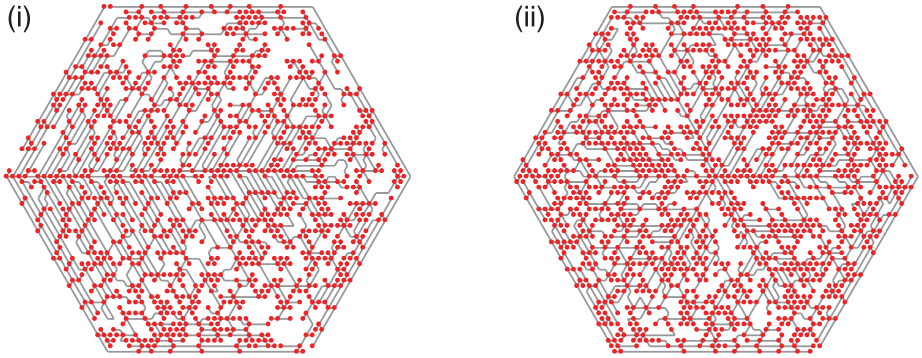

Fig C. Examples of the unweighted structure of simulated optimal distribution networks. Model networks were constructed by optimizing transport in the presence of fluctuations in load. *(i)* An example of a simulated transport network in which the source was placed at the left edge. *(ii)* An example of a simulated transport network in which the source was placed at the center. In both panels, gray lines correspond to network edges and red circles correspond to network nodes.

Generating optimal transport networks

In addition to analyzing empirical distribution networks, another important direction is understanding what kinds of construction rules or theoretical models for transport networks could give rise to organizational principles observed in real data, such as hierarchical structure, for example [4]. It has been shown in the past that many traits of organisms in general, and biological transport networks in particular, can be explained by the assumption that they are optimized for their function in some sense. In the case of transport networks such as leaf venation or mammalian capillary networks, one expects that they minimize the dissipated power in the network while at the same time being robust against external damage such as embolisms or vein occlusions [4]. A developmental mechanism that can produce optimized networks using local adaptive rules was discussed in [5]. In the remainder of this section, we review the model of optimal transport networks from [4] that is able to produce realistic efficient and robust network topologies. Then in Rentian scaling analysis we investigate the model networks for Rentian scaling, noting that the goal of the analysis is not to capture specific details and differences in each family of networks, but rather to shed light on a common mechanism that may be able to reproduce some of the key spatial and topological properties that are present in *both* systems.

We begin by noting that blood flow in the smallest capillaries can be modeled by laminar flow in cylindrical vessels of radius R according to Poiseuille's law. Volume flow is then $Q = K\Delta p$, where the conductivity is $K = \frac{\pi}{8\mu L}R^4$, L is vessel length, μ is fluid viscosity, and Δp is the pressure drop along the vessel. The capillary network itself is modeled as a graph with N nodes where each

edge (i, j) carries a volume flow Q_{ij}. At each node i we have conservation of current,

$$\sum_j Q_{ij} = q_i, \tag{5}$$

where q_i is the perfusion rate, or net current drawn from the network at the node. We note that Eq. 5 can be solved for the volume flows Q_{ij} given the conductivities K_{ij} and the net currents q_i.

Next, we note that an optimal network minimizes the dissipated power during operation, which is defined by

$$P = \sum_{(ij)} \frac{Q_{ij}^2}{K_{ij}}. \tag{6}$$

To force the network to be robust in the presence of fluctuating load, [4] introduced the load-averaged power dissipation,

$$\langle P \rangle = \sum_{(ij)} \frac{\langle Q_{ij}^2 \rangle}{K_{ij}}. \tag{7}$$

Here, the angle brackets $\langle f \rangle = \frac{1}{N-1} \sum_k f^{(k)}$ denote an average over all possible network configurations with net currents

$$q_i^{(k)} = \delta_{ii_0} - \delta_{ik}, \tag{8}$$

where we fix the source i_0 and vary the sink $k \in \{1, \ldots, N\} \setminus \{i_0\}$. This is called the moving sink model. Finally, we note that a well-posed optimization problem requires the introduction of a constraint that prevents the conductivities from diverging. A biologically sensible constraint is to set

$$\sum_{(ij)} K_{ij}^{\frac{1}{2}} = const. \tag{9}$$

In this way, the total volume of the network is kept fixed, which can be interpreted as the organism having a finite amount of resources at its disposal to construct its venation network.

The resulting constrained optimization problem is then solved using a simulated annealing algorithm as in [4]. As the initial condition we choose a triangular lattice network with randomly initialized edge conductivities. The net currents q_i are chosen to represent one source either at one edge of the lattice or at the center, and uniform sinks everywhere else, modeling uniform perfusion requirements. Example networks generated from placing the source node at one edge or in the center are shown in Fig. C(i) and Fig. C(ii), respectively.

After optimization, the result is a weighted network, where the weights correspond to the conductance between pairs of connected nodes. For each source

location (edge or center), we constructed an ensemble of 10 simulated networks (20 in total). All conductances below a threshold value of 3.5×10^{-8} were set to zero. Furthermore, since we wish to investigate these networks for Rentian scaling, which depends only on the binary connectivity of a graph (and on edge and node locations for physical scaling), after thresholding, we binarized each network by setting all remaining non-zero conductances to unity. This resulted in a set of unweighted adjacency matrices in the same form as the empirical data. Further details on the Rentian scaling analysis are provided in the section below.

Rentian scaling analysis

In the main text, we examined the vasculature and mycelial networks for Rentian scaling. Fig. D below shows schematics demonstrating how to compute topological (*top row*) and physical (*bottom row*) Rentian scaling on a network, and we provide additional details on these methods in the following two sections.

Additional details and figures for topological Rentian scaling

To test for the presence of topological Rentian scaling (see Fig. D(i),(ii) below) in the biological distribution networks, we employed a min-cut bi-partitioning algorithm that attempts to recursively partition a network into halves, quarters, etc., while minimizing the number of edges cut during the bi-sectioning process. At each level of partitioning, the algorithm records the mean number of nodes n in the topological partitions at the given level, and the mean number of edges m crossing the boundaries of the partitions at the given level. Topological Rentian scaling exists if a relationship of the form $m \propto n^t$ holds across several partition sizes. It is important to point out that because the problem of finding a minimum-cut bi-section is NP-complete, Rentian scaling is a *heuristic* [6], and the resulting values of the exponents may to some extent be procedure dependent. In this work we utilized the hyper-graph partitioning package *hMETIS* [7,8] to compute topological scaling (*vs.* a spectral approach, for example), because it is considered a high quality and fast algorithm, and has been used in previous studies to determine scaling exponents of different kinds of complex networks [7,9–11].

Another important point of consideration in this analysis is the occurrence of boundary effects due to large partitions and the finite size of the networks under consideration. In particular, for the topological scaling, large partitions (i.e., on the same scale as the network size) can exhibit large reductions in the number of crossing edges relative to the number of nodes inside, thereby skewing the scaling relationship. In the VLSI literature, this drop-off in the power law relationship between m and n is termed "Region II" [12,13] (see Fig. E). In line with common practice, we thus estimated the scaling exponent t from "Region I", where the partition sizes are typically small enough to avoid being biased by

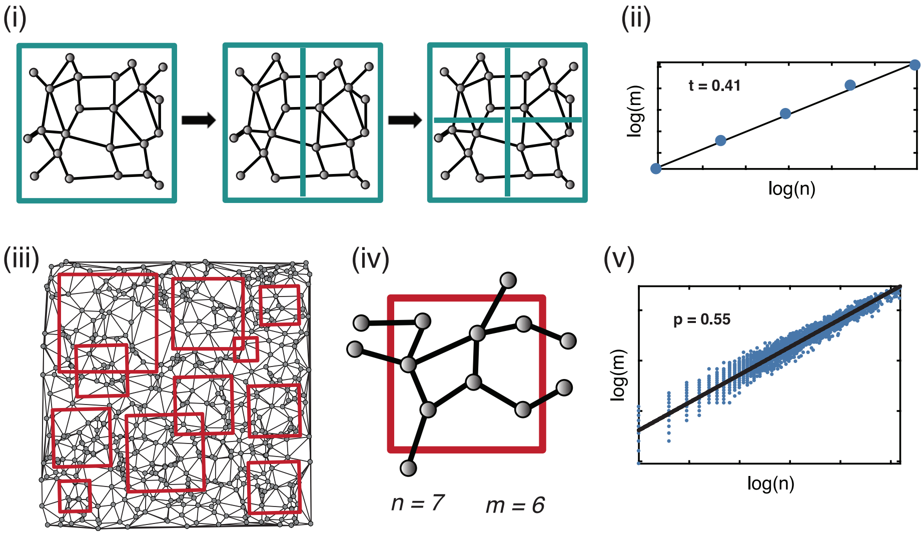

Fig D. Schematics of topological and physical Rentian scaling. *(Top row)* An example of topological Rentian scaling. *(i)* A schematic of the topological partitioning process on a toy network. Beginning with a single box that covers the entire network, we recursively partition the network into halves, quarters, etc., such that the number of nodes n within each partition is roughly equal, and the number of edges m that cross partitions is minimum. *(ii)* If topological scaling exists, n and m scale with one another in $\log - \log$ space and the topological Rent exponent t is given by the slope of the best-fit line. *(Bottom row)* An example of physical Rentian scaling. *(iii)* We test for Rentian scaling in real space by placing boxes of varying length scales at random over the network. *(iv)* For each box, we count the number of nodes inside n and the number of edges crossing the boundary m, and consider how these quantities scale in $\log - \log$ space. *(v)* If scaling exists, n and m scale with one another in $\log - \log$ space and the physical Rent exponent p is determined from the slope of the best fit line.

boundary effects. For consistency across all networks, we always neglected the first three levels of partitioning (see Fig. E).

An additional methodological consideration is that *hMETIS* is not guaranteed to converge to an optimal solution, and therefore, different runs of the algorithm (for the same network) may yield slightly different results. In order to take this variation into account, we ran the partitioning 50 times for all networks considered, and reported average values of relevant quantities over the 50 trials. In particular, for each run of the algorithm on a given network, we computed a simple linear regression (using MATLAB's 'polyfit' function) on $\log m$ *vs.* $\log n$, and examined the mean coefficient of determination r^2 over the 50 trials as a way to quantify the goodness of the fit. We found all $r^2 > 0.987$ for the set of mycelial networks and all $r^2 > 0.981$ for the set of vasculature networks. These

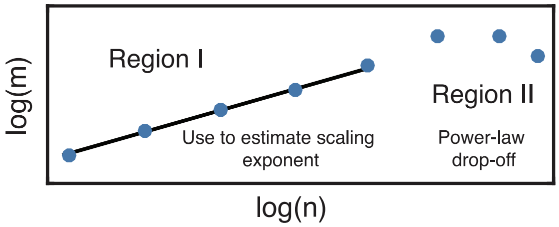

Fig E. A schematic of "Region I" and "Region II" behavior in Rentian scaling. Since the networks have a finite size, large topological partitions are subject to boundary effects that cause a drop-off in the power-law scaling ("Region II"). Therefore, in order to assess networks for topological scaling, we neglected the largest three partitions, and used the data in "Region I". If scaling exists, these points exhibit a linear relationship in $\log - \log$ space, and the topological exponent can be estimated from the slope of a best-fit line through the data.

results indicated that the empirical networks display relatively good topological Rentian scaling. After each run of the partitioning on a given network, we also estimated the topological scaling exponent from the slope of a best-fit line to $\log m$ *vs.* $\log n$ (also computed using MATLAB's 'polyfit' function). Averaged over the 50 runs of the partitioning algorithm, we found that the topological Rent exponent t ranged between 0.234 and 0.322 for the set of mycelial networks, and between 0.122 and 0.248 for the set of vasculature networks.

We showed examples in the main text of the topological scaling relationship for a mycelial network from *P.V. 1* (Fig. 5A) and for a rat vasculature network (Fig. 5B). Here, we additionally provide examples of $\log m$ *vs.* $\log n$ for the other types of fungi – *P.I.*, *P.V. 2*, and *R.B.* – in Fig. F(i)–(iii), and for a mouse vasculature network in Fig. F(iv).

We also examined the simulated transport networks developed from biologically inspired optimization principles [4, 14–16] for the presence of topological Rentian scaling. In particular, we constructed 10 model networks in which the source was placed at one edge and 10 model networks in which the source was placed at the center (see Generating optimal transport networks for details on the model construction and simulations). To first test each ensemble of simulations for the presence of topological Rentian scaling, we ran 50 runs of the topological partitioning on each network, and used MATLAB's 'polyfit' function to compute a simple linear regression on $\log m$ *vs.* $\log n$ for each of those runs. Averaged over the 50 trials, we found all $r^2 > 0.985$ for the set of networks with the source at one edge, and we found all $r^2 > 0.992$ for the set of networks with the source at the center. These results suggested that topological Rentian scaling is present in the simulated optimal transport networks as well. The topological Rent exponents were determined from the slope of a best-fit line to $\log m$ *vs.* $\log n$, and we considered mean values of the exponent t averaged over the 50 runs of the partitioning algorithm. For the set of networks with the source at one edge, the (averaged) topological Rent exponent ranged between

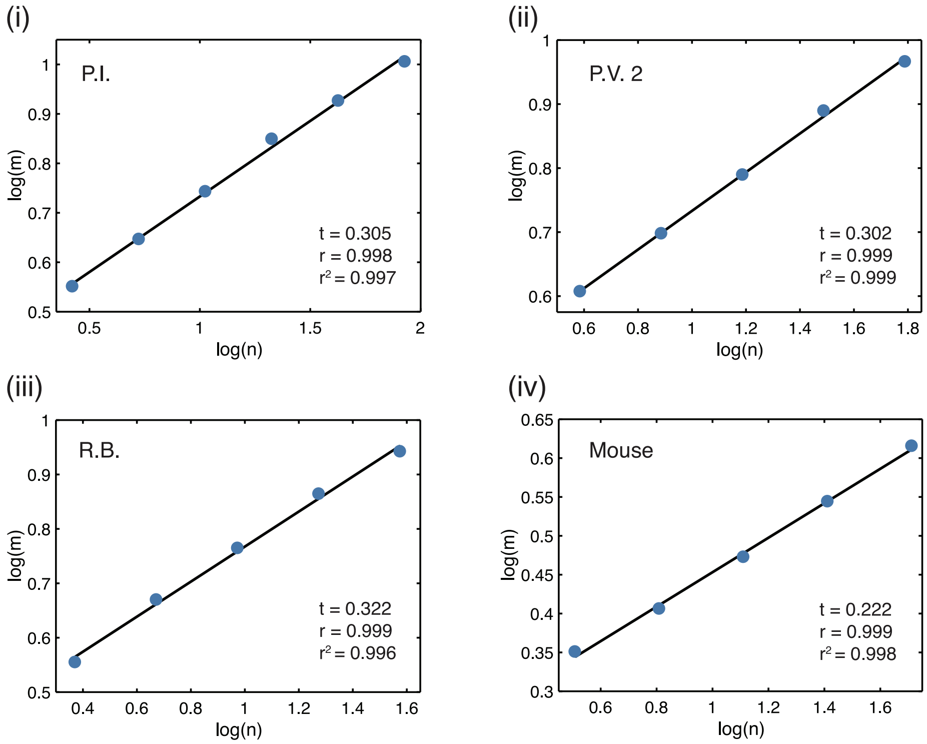

Fig F. Additional examples of topological Rentian scaling in biological distribution networks. To test for the presence of this phenomena, we recursively sectioned each network into halves, quarters, etc., and counted the mean number of nodes n inside each partition at a given level and the mean number of edges m crossing the boundary of each partition at a given level. Rentian scaling exists if these quantities scale with one another in $\log - \log$ space, and the topological Rent exponent t can be estimated from the slope of a best fit line through $\log m$ *vs.* $\log n$. Examples of $\log m$ *vs.* $\log n$ for *(i)* a mycelial network from *P.I.*, *(ii)* a mycelial network from *P.V. 2*, *(iii)* a mycelial network from *R.B.*, and *(iv)* an arterial network from a mouse brain. In each panel, the black lines correspond to a best-fit line through the data, from which we estimated the displayed scaling exponent t, the Pearson correlation coefficient r, and the coefficient of determination r^2.

$t = 0.196$ and $t = 0.262$, and for the set of networks with the source at the center, the (averaged) topological Rent exponent ranged between $t = 0.233$ and $t = 0.311$. Fig. G(i),(ii) show examples of $\log m$ *vs.* $\log n$ from one run of the topological partitioning process on a simulated network with the source at one edge and with the source at the center, respectively.

In order to compare and contrast topological Rentian scaling across different kinds of networks, we normalized the topological Rent exponents t of the empirical and model networks by their mean values $\langle t_{\text{rewire}} \rangle$ from each networks'

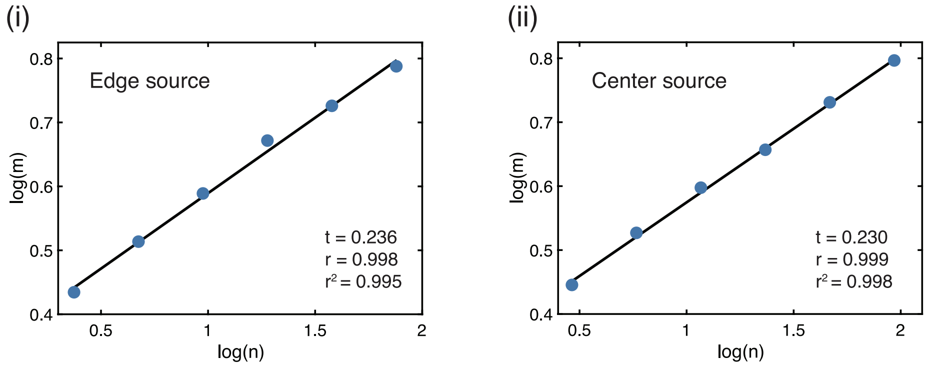

Fig G. Examples of topological Rentian scaling in simulations of optimal transport networks. To test for the presence of this phenomena, we recursively sectioned each network into halves, quarters, etc., and counted the mean number of nodes n inside each partition at a given level and the mean number of edges m crossing the boundary of each partition at a given level. Rentian scaling exists if these quantities scale with one another in $\log - \log$ space, and the topological Rent exponent t can be estimated from the slope of a best fit line through $\log m$ *vs.* $\log n$. Examples of $\log m$ *vs.* $\log n$ for *(i)* a simulated network in which the source was placed at one edge, and *(ii)* a simulated network in which the source was placed in the center. In both panels, the black lines correspond to a best fit line through the data, from which we estimated the displayed scaling exponent t, the Pearson correlation coefficient r, and the coefficient of determination r^2.

set of randomly-rewired null models (see Null models in the main text). To be concrete, $\langle t_{\text{rewire}} \rangle$ was computed as follows. For each of the 10 instantiates of the randomly-rewired null-model networks (corresponding to either a single empirical or a single simulated network), we ran the topological partitioning 50 times and computed the mean scaling exponent t_{rewire} over those 50 trials. We then averaged t_{rewire} over the set of 10 null-model networks to obtain a single value $\langle t_{\text{rewire}} \rangle$.

Additional details and figures for physical Rentian scaling

We next wished to investigate whether or not the empirical data showed evidence of physical Rentian scaling, i.e., a relationship of the form $m \propto n^p$, where m is the number of edges crossing a physical partition of the network, n is the number of nodes inside the partition, and p is the physical Rent exponent (see Fig. D(iii)–(v)). We quantified this relation via consideration of the coefficient of determination r^2, computed from a simple linear regression (using MATLAB's 'polyfit' function) on $\log m$ *vs.* $\log n$ from 5000 spatial partitions of the network. As with the estimation of topological scaling, in order to obtain a good estimate of physical Rentian scaling throughout the majority of the network, one should be cognizant of the finite size of the system. Because boxes placed close to

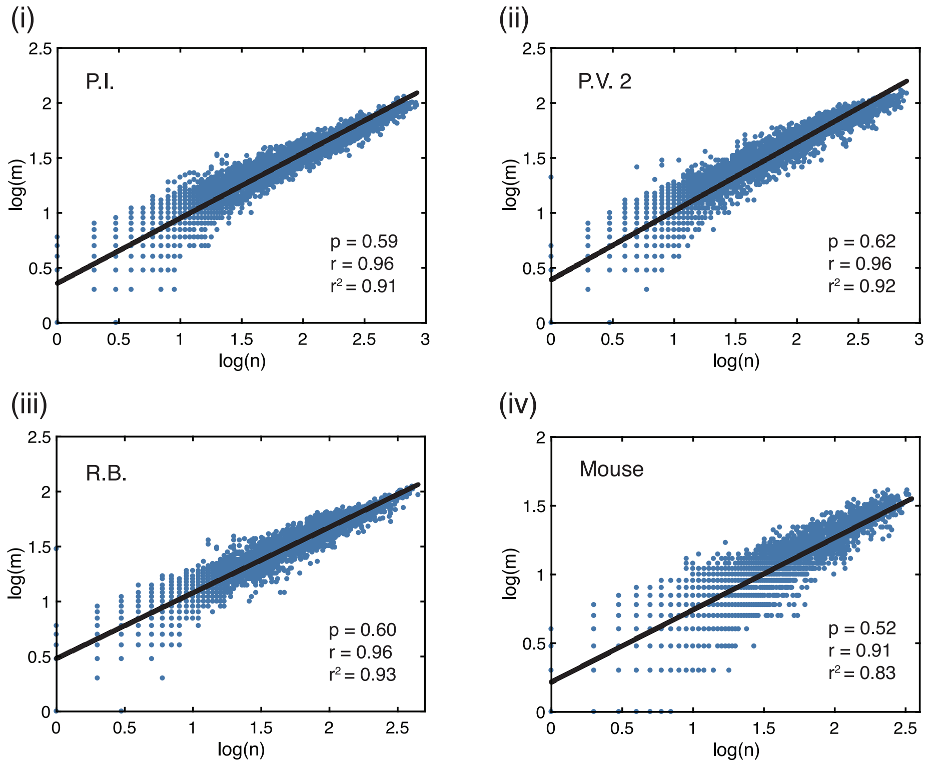

Fig H. Additional examples of physical Rentian scaling in biological distribution networks. To test for the presence of this phenomena in real space, we partitioned each network into 5000 boxes of varying side lengths and center positions, and counted the number of nodes inside n and the number of edges crossing the boundary m of each box. Rentian scaling exists if these quantities scale with one another in $\log - \log$ space, and the physical Rent exponent p can be determined from the slope of a best fit line through $\log m$ *vs.* $\log n$. Examples of $\log m$ *vs.* $\log n$ for *(i)* a mycelial network from *P.I.*, *(ii)* a mycelial network from *P.V. 2*, *(iii)* a mycelial network from *R.B.*, and *(iv)* an arterial network from a mouse brain. The lines correspond to a best fit line through the data, from which we estimated the displayed scaling exponent p, the Pearson correlation coefficient r, and the coefficient of determination r^2.

the boundary of the network will contain significantly fewer outgoing edges than boxes placed within the bulk, we only included boxes within the network's convex hull to try and mitigate this issue. For the mycelial networks, we found all $r^2 > 0.88$ and for the vasculature networks, we found all $r^2 > 0.82$. These values indicate relatively good fits and suggest the presence of physical Rentian scaling in the data. In addition to testing each network for the existence of physical Rentian scaling, our second aim was to estimate values of the physical Rent exponent in order to quantify and characterize the network architecture, and to compare across networks. Physical Rent exponents p were estimated from the slope of a best fit line (computed using MATLAB's 'polyfit' function) to $\log m$ *vs.* $\log n$. For the mycelial networks, the physical Rent exponents ranged between $p = 0.57$ and $p = 0.64$, and for the vasculature networks, the physical Rent exponents ranged between $p = 0.52$ and $p = 0.54$. In the main text, we showed examples of $\log m$ *vs.* $\log n$ for a mycelial network from *P.V. 1* (Fig. 5C) and for a rat vasculature network (Fig. 5D). Here, we also show explicit examples of this relationship for the other types of fungi – *P.I.*, *P.V. 2*, and *R.B.* – in Fig. H(i)–(iii), and for a mouse vasculature network in Fig. H(iv).

We also examined Rentian scaling in the ensemble of simulated model networks (see Generating optimal transport networks). More specifically, we constructed 10 model networks using a source at one edge and 10 model networks using a source at the center, and first tested for the presence of physical Rentian scaling in each ensemble. Using MATLAB's 'polyfit' function, we computed a simple linear regression on $\log m$ *vs.* $\log n$. For the set of networks with the source at one edge, we found all $r^2 > 0.84$, and for the set of networks with the source at the center, we found all $r^2 > 0.89$. These results suggested that the model networks also exhibit evidence of physical Rentian scaling. The physical Rent exponents were determined from the slope of the best-fit line for $\log m$ *vs.* $\log n$. For the set of networks with the source at one edge, the physical Rent exponents ranged between $p = 0.54$ and $p = 0.56$, and for the set of networks with the source at the center, the physical Rent exponents ranged between $p = 0.54$ and $p = 0.57$. Fig. I(i),(ii) show examples of $\log m$ *vs.* $\log n$ computed using physical partitions of a simulated network with the source at one edge and with the source at the center, respectively.

To allow for a cleaner comparison across different networks, we normalized the physical Rent exponents of the empirical and model networks by the mean physical Rent exponent $\langle p_{\text{rewire}} \rangle$ of each networks' set of randomly-rewired null models (see Null models in the main text). The randomly-rewired null model networks were analyzed for physical Rentian scaling in the same manner described above.

Fig. J shows the topological Rent exponent ratio $\tilde{t} = t/\langle t_{\text{rewire}} \rangle$ *vs.* the physical Rent exponent ratio $\tilde{p} = p/\langle p_{\text{rewire}} \rangle$ for both the empirical and simulated networks. We observe that the physical exponent ratios from the simulated networks lie approximately in the center of the physical exponent ratios from the real data. The topological exponent ratios from the simulated networks are also in the same general vicinity as the empirical topological exponent ratios,

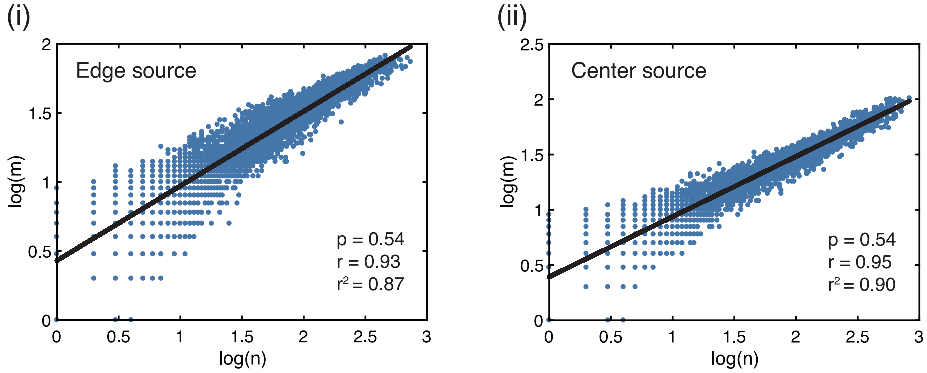

Fig I. Examples of physical Rentian scaling in simulations of optimal transport networks. To test for the presence of this phenomena in real space, we partitioned each network into 5000 boxes of varying side lengths and center positions, and counted the number of nodes inside n and the number of edges crossing the boundary m of each box. Rentian scaling exists if these quantities scale with one another in $\log-\log$ space, and the physical Rent exponent p can be determined from the slope of a best fit line through $\log m$ *vs.* $\log n$. Examples of $\log m$ *vs.* $\log n$ for *(i)* a simulated network in which the source was placed at one edge, and *(ii)* a simulated network in which the source was placed in the center. The lines correspond to a best fit line through the data, from which we estimated the displayed scaling exponent p, the Pearson correlation coefficient r, and the coefficient of determination r^2.

although they tend to be closer to the values observed in the mycelial networks and are smaller than those from several of the vasculature networks. These results suggest that the optimization of transport under fluctuations provides a mechanism able to produce simulated, model networks with some important architectural properties (e.g. hierarchical organization) similar to the empirical data.

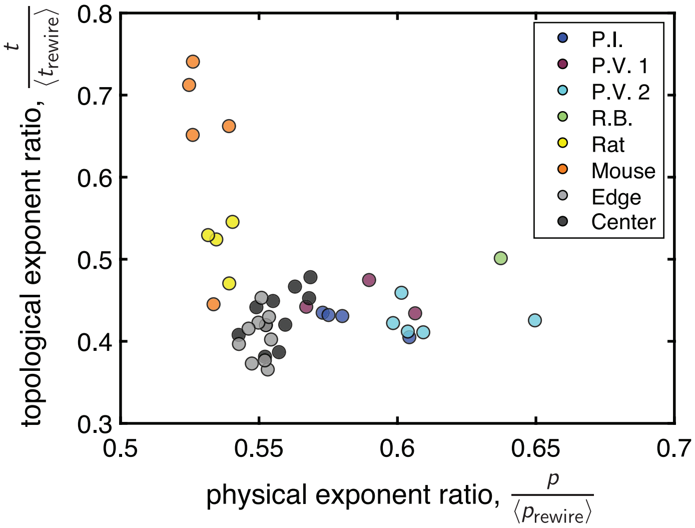

Fig J. The topological Rent exponent ratio *vs.* the physical Rent exponent ratio for both the empirical (colored circles) and simulated transport networks (gray cirlces).

Additional analysis of network robustness

In the main text, we used random edge removal to probe the structural robustness of each network. However, it can also be useful to consider ordered edge removal that is based on an edge property. For example, studies on mycelial networks suggest that edge removal based on combinations of cord length and radii are more biologically relevant [17]. Although we do not have cross-sectional area information for the vasculature network vessels, we can still carry out edge-removal based on link length for both the vasculature and mycelial networks.

We computed robustness curves when edge removal was ordered by the physical length of links (specifically, edges were removed in order of longest to shortest). Fig. K shows three different variations of this analysis: panel *(i)* displays the fraction of nodes in the largest connected component *vs.* the fraction of total edges removed, panel *(ii)* displays the fraction of total wiring length in the largest connected component *vs.* the fraction of total edges removed, and panel *(iii)* displays the fraction of total wiring length in the largest connected component *vs.* the fraction of total wiring length removed from the network.

For each variation, we observe that in general, the vasculature networks tend to fall apart more rapidly than the majority of the mycelial networks. This analysis supports the findings in the main text using the spatial null models and random edge removal.

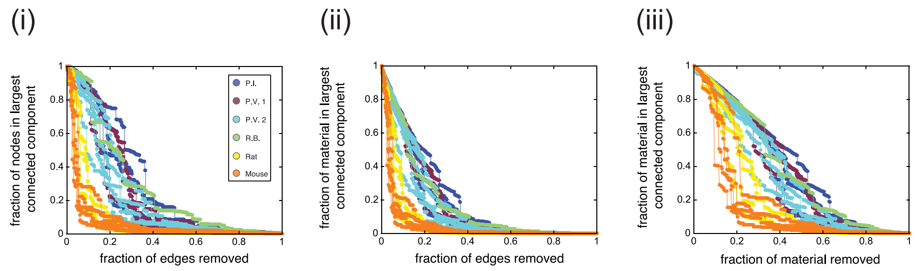

Fig K. Structural robustness curves for ordered edge removal based on the physical length of links. For each set of curves, edges were removed from longest to shortest. *(i)* The fraction of edges removed *vs.* the fraction of nodes in the largest connected component. *(ii)* The fraction of edges removed *vs.* the fraction of total wiring length in the largest connected component. *(iii)* The fraction of total wiring length removed *vs.* the fraction of total wiring length in the largest connected component.

Examination of vasculature networks without penetrating arteriole offshoots

For the vasculature systems, it is important to note that nodes are defined either as junctions where surface vessels merge or points where penetrating arterioles delve into the underlying tissue. Moreover, while some penetrating arterioles originate *en passant*, many of them exist at one end of a surface vessel offshoot [18]. Though the presence of this latter type of penetrating arteriole is a meaningful architectural feature of the vasculature that we opted to incorporate into our primary analysis, it is also of interest to understand if and how results change when the main surface network is analyzed without the penetrating arterioles that reside at one end of a surface vessel. In order to examine this, we iteratively removed all penetrating arterioles with degree one (and the corresponding edges) from the vasculature networks. This process results in a "reduced" network that contains all of the nodes that were part of the original surface network (that is, those nodes resulting from a branch point among surface vessels), but that does not contain the isolated penetrating arteriole offshoots. Fig. L(i) shows an example of a full mouse vasculature network with surface nodes shown in red and penetrating arterioles shown in blue, and Fig. L(ii) shows the corresponding reduced network after recursively removing the degree-one penetrating arterioles. (Note that the reduced network still contains *en passant* penetrating arterioles that exist in between surface

network nodes). Crucially, the reduced networks generated in this way are subgraphs of the original system, and furthermore, all edges (and nodes) in the reduced networks have the same physical locations they did in the original network.

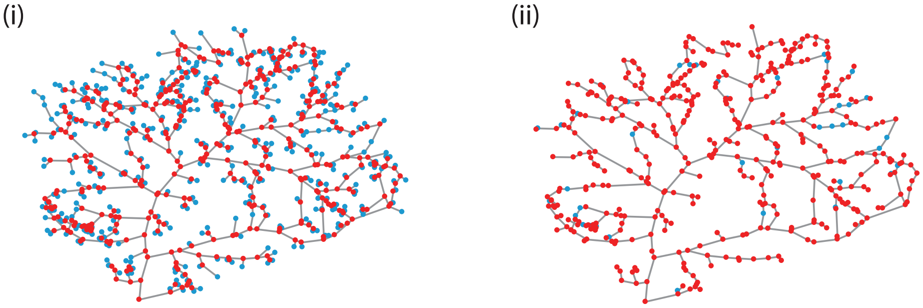

Fig L. Construction of reduced networks without penetrating arteriole offshoots. *(i)* An example of a vasculature network from a mouse brain. Surface vessel segments are represented as edges (gray lines) and merge to form branching points on the surface (red nodes) or connect to penetrating arterioles that dive into the tissue (blue nodes). *(ii)* The corresponding reduced network, generated by iteratively removing penetrating arterioles of degree one from the original network. A network analysis can be carried out on both the original and reduced networks, and results can be compared across the two cases.

To test whether results are consistent when considering the reduced rather than the complete networks, we performed two analyses: *(1)* a comparison of each network measure between the full and reduced networks, and *(2)* a comparison of each network measure between the reduced vasculature networks and the mycelial networks. The first analysis allows us to understand how network organization differs between the full and reduced systems, and the second analysis allows us to examine whether the results reported for the comparison of the vasculature and mycelial networks still hold when the mycelial networks are contrasted against the reduced vasculature networks.

Because the total number of networks is small, in order to enhance statistical power we first combined the original rat and mouse networks into one set and the reduced rat and mouse networks into another set. For every network in the two groups ("Full Vasculature" and "Reduced Vasculature") we then computed the network measures defined in the Network measures section of the main text and summarized in Table 2 of the main text. To compare the full networks to the reduced networks, we performed paired-sample t-tests on the data. In particular, a statistically significant difference in the mean value of a given network property between the two groups is indicated by a p-value < 0.05. The results of this first examination are compiled in Table A, where the first column indicates the network property under consideration, and the second and third columns display the mean $\pm$ the standard error of the mean of the given

property computed on the set of full vasculature networks and the set of reduced vasculature networks, respectively. A star (*) to the right of the value in the "Reduced Vasculature" column indicates that there was a statistically significant difference in the corresponding network property between the full *vs.* reduced networks. We found small albeit significant differences in the following measures: mean degree $\langle k \rangle$, alpha index α, Spearman correlation ρ between topological and physical edge betweenness centrality, ratio of theoretical minimum physical Rent exponent to true physical Rent exponent $p^{\min}/p$, relative wiring W_{rel}, relative physical efficiency E_{rel}, and relative robustness R_{rel}. However, as detailed below, we show that, importantly, these minor changes in network properties do not change the conclusions of the comparison between the vasculature and mycelial networks.

Turning now to the second analysis, we compare the mycelial networks to the reduced vasculature networks in which penetrating arteriole offshoots are removed. This investigation was carried out equivalently to the analysis in the main text, but the full vasculature networks were replaced by their reduced versions. We summarize the results in Table B, where the first column indicates the network property under consideration, the second and third columns give the mean $\pm$ the standard error of the mean for each network property (averaged over the population of mycelial networks and the population of reduced vasculature networks, respectively), and the last column indicates the level of significance from a two-sample t-test used to assess statistical differences in the mean values of each network property for the two classes of distribution networks; **p-value < 0.001, ***p-value < 0.0001.

We found that all differences between the mycelia and vasculature that were statistically significant when considering the full vasculature networks remain significant when using the reduced vasculature networks instead. Thus, the major conclusions from the investigation in the main text are robust with respect to whether the full or reduced vasculature networks are used in the analysis. Aside from the fact that this finding demonstrates a general robustness of results, it is also useful because it elucidates the fact that, although some measures change by a statistically significant amount in going from the full to the reduced networks, these differences are not enough to overwhelm the distinctions between the vasculature and mycelia. In other words, differences between the vasculature and mycelia cannot be explained solely by the fact that the vasculature systems have a number of penetrating arterioles at the ends of surface vessels.

Table A. Comparison of network measures between the full and reduced vasculature networks. The first column indicates the network measure under consideration and the second and third columns indicate the mean ± the standard error of the mean of the given measure computed over the population of the full vasculature networks and the reduced vasculature networks, respectively. A star (*) to the right of the reduced value indicates that there was a statistically significant difference (as determined by a paired-sample t-test with p-value < 0.05) between the value of the given measure in the full *vs.* reduced version of the network. No star indicates that there was not a statistically significant difference.

Network Property	Full Vasculature	Reduced Vasculature
Mean degree $\langle k \rangle$	2.09 ± 0.01	2.15 ± 0.02 *
Normalized clustering $\frac{C}{\langle C_{\text{rewire}} \rangle}$	6.44 ± 1.61	9.78 ± 6.14
Alpha index α	0.022 ± 0.003	0.039 ± 0.004 *
Normalized topological efficiency $\frac{E^{\text{t}}}{\langle E^{\text{t}}_{\text{rewire}} \rangle}$	0.84 ± 0.03	0.84 ± 0.03
Spearman correlation between topological and physical edge betweenness centrality ρ	0.94 ± 0.01	0.85 ± 0.02 *
Normalized physical Rent exponent $\frac{p}{\langle p_{\text{rewire}} \rangle}$	0.533 ± 0.002	0.522 ± 0.004 *
Normalized topological Rent exponent $\frac{t}{\langle t_{\text{rewire}} \rangle}$	0.59 ± 0.04	0.56 ± 0.03
Ratio of theoretical minimum physical Rent exponent to true physical Rent exponent $\frac{p^{\text{min}}}{p}$	0.949 ± 0.004	0.978 ± 0.008 *
Relative wiring W_{rel}	0.080 ± 0.004	0.067 ± 0.005 *
Relative physical efficiency E_{rel}	0.36 ± 0.03	0.41 ± 0.03 *
Relative robustness R_{rel}	0.15 ± 0.01	0.16 ± 0.01 *

Table B. Comparison of network measures between the mycelial and the reduced vasculature networks. The first column indicates the network property. The second and third columns give the mean ± the standard error of the mean for each network property averaged over the population of mycelial networks and the population of reduced vasculature networks, respectively. The last column indicates the level of significance from a two-sample t-test used to assess statistical differences in the mean values of each network property between the two types of distribution networks; **p-value < 0.001, ***p-value < 0.0001.

Network property	Mycelial fungi	Vasculature (Reduced)	Significance level
Mean degree $\langle k \rangle$	2.57 ± 0.05	2.15 ± 0.02	***
Normalized clustering $\frac{C}{\langle C_{\mathrm{rewire}} \rangle}$	54.67 ± 7.88	9.78 ± 6.14	**
Alpha index α	0.144 ± 0.012	0.039 ± 0.004	***
Normalized topological efficiency $\frac{E^{\mathrm{t}}}{\langle E^{\mathrm{t}}_{\mathrm{rewire}} \rangle}$	0.60 ± 0.02	0.84 ± 0.03	***
Spearman correlation between topological and physical edge betweenness centrality ρ	0.65 ± 0.03	0.85 ± 0.02	***
Normalized physical Rent exponent $\frac{p}{\langle p_{\mathrm{rewire}} \rangle}$	0.609 ± 0.007	0.522 ± 0.004	***
Normalized topological Rent exponent $\frac{t}{\langle t_{\mathrm{rewire}} \rangle}$	0.44 ± 0.01	0.56 ± 0.03	**
Ratio of theoretical minimum physical Rent exponent to true physical Rent exponent $\frac{p^{\mathrm{min}}}{p}$	0.826 ± 0.007	0.978 ± 0.008	***
Relative wiring W_{rel}	0.302 ± 0.013	0.067 ± 0.005	***
Relative physical efficiency E_{rel}	0.43 ± 0.03	0.41 ± 0.03	—
Relative robustness R_{rel}	0.38 ± 0.02	0.16 ± 0.01	***

References

1. Watts DJ, Strogatz SH. Collective dynamics of 'small-world' networks. Nature. 1998;393(6684):440–442.

2. Barthélemy M. Spatial networks. Phys Rep. 2011;499(1–3):1–101.

3. Latora V, Marchiori M. Efficient Behavior of Small-World Networks. Phys Rev Lett. 2001;87(19):198701.

4. Katifori E, Szöllősi GJ, Magnasco MO. Damage and fluctuations induce loops in optimal transport networks. Phys Rev Lett. 2010;104(4):048704.

5. Ronellenfitsch H, Katifori E. Global optimization, local adaptation, and the role of growth in distribution networks. Phys Rev Lett. 2016;117(13):138301.

6. Hagen L, Kahng AB, Kurdahi FJ, Ramachandran C. On the Intrinsic Rent Parameter and Spectra-Based Partitioning Methodologies. IEEE Transactions on Computer-Aided Design of Integrated Circuits and Systems. 1994;13(1):27–37.

7. Karypis G, Aggarwal R, Kumar V, Shekhar S. Multilevel hypergraph partitioning: applications in VLSI domain. IEEE Transactions on Very Large Scale Integration (VLSI) Systems. 1999;7(1):69–79.

8. hMETIS - Hypergraph & Circuit Partitioning. Available from: `http://glaros.dtc.umn.edu/gkhome/metis/hmetis/overview`.

9. Bassett DS, Greenfield DL, Meyer-Lindenberg A, Weinberger DR, Moore SW, Bullmore ET. Efficient Physical Embedding of Topologically Complex Information Processing Networks in Brains and Computer Circuits. PLoS Comput Biol. 2010;6(4):1–14.

10. Klimm F, Bassett DS, Carlson JM, Mucha PJ. Resolving structural variability in network models and the brain. PLoS Comput Biol. 2014;10(3):1–22.

11. Sperry MM, Telesford QK, Klimm F, Bassett DS. Rentian scaling for the measurement of optimal embedding of complex networks into physical space. Journal of Complex Networks. 2016;5(2):199–218.

12. Greenfield DL. Communication Locality in Computation: Software, Chip Multiprocessors and Brains. University of Cambridge; 2010.

13. Christie P, Stroobandt D. The interpretation and application of Rent's rule. IEEE Transactions on Very Large Scale Integration (VLSI) Systems. 2000;8(6):639–648.

14. Banavar JR, Colaiori F, Flammini A, Maritan A, Rinaldo A. Topology of the Fittest Transportation Network. Phys Rev Lett. 2000;84(20):4745–4748.

15. Bohn S, Magnasco MO. Structure, Scaling, and Phase Transition in the Optimal Transport Network. Phys Rev Lett. 2007;98(8):088702.

16. Durand M. Structure of optimal transport networks subject to a global constraint. Phys Rev Lett. 2007;98(8):088701.

17. Bebber DP, Hynes J, Darrah PR, Boddy L, Fricker MD. Biological solutions to transport network design. Proceedings of the Royal Society of London B: Biological Sciences. 2007;274(1623):2307–2315.

18. Blinder P, Shih AY, Rafie C, Kleinfeld D. Topological basis for the robust distribution of blood to rodent neocortex. Proceedings of the National Academy of Sciences. 2010;107(28):12670–12675.
